# Causal Relationships Between Major Depressive Disorder and Coronary Artery Disease Across Diverse Populations: A Bidirectional Mendelian Randomisation Study

**DOI:** 10.1111/tmi.70051

**Published:** 2025-10-15

**Authors:** Sarah Silva, Dorothea Nitsch, Segun Fatumo

**Affiliations:** ^1^ Department of Non‐Communicable Disease Epidemiology London School of Hygiene & Tropical Medicine London UK; ^2^ The African Computational Genomics Research Group (TACG), MRC/UVRI and LSHTM Entebbe Uganda; ^3^ Precision Healthcare University Research Institute, Queen Mary University of London London UK

**Keywords:** bidirectional two‐sample Mendelian randomisation analysis, coronary artery disease, coronary heart disease, depression, major depressive disorder, Mendelian randomisation, multi‐ancestry

## Abstract

**Background and Objectives:**

Coronary artery disease remains the leading cause of cardiovascular mortality worldwide, with a disproportionate burden in low‐ and middle‐income countries. Although observational studies have established a bidirectional relationship between depression and coronary artery disease, the underlying genetic basis of this association remains unclear, particularly in underrepresented diverse‐ancestry populations. Establishing whether this relationship is causal and whether it differs by ancestry is critical for informing targeted and equitable prevention strategies.

**Methodology:**

This study employed a bidirectional two‐sample Mendelian randomisation framework to investigate the causal relationship between major depressive disorder and coronary artery disease across East Asian, European and African populations. Using summary statistics from large‐scale genome‐wide association studies, we assessed both the direction and strength of genetic associations between major depressive disorder and coronary artery disease. Sensitivity analyses were conducted to test the robustness of the findings.

**Results:**

Genetically predicted major depressive disorder was associated with increased coronary artery disease risk in European populations, confirming a causal effect consistent with prior evidence. In East Asians, we found a potentially false‐positive result suggesting an inverse association between genetic liability to coronary artery disease and major depressive disorder (IVW OR = 0.942; *p* = 0.040), which was not supported across Mendelian randomisation sensitivity analyses and did not hold true after Bonferroni correction. In African ancestry populations, no significant causal effects were detected in either direction, due to wide confidence intervals resulting from limited sample sizes.

**Conclusions:**

Our findings provide evidence for a causal relationship between depression and coronary artery disease in Europeans and suggest potential ancestry‐specific variation in this relationship, with a novel inverse association between coronary artery disease and major depressive disorder observed in East Asians requiring further replication and validation. These results highlight how limited data availability in underrepresented populations constrains causal inference, underscoring the need for more inclusive genetic research to inform equitable, population‐relevant public health strategies.

## 1 Introduction

Globally, coronary artery disease (CAD) alone accounts for 42% of cardiovascular disease (CVD)‐related deaths, placing immense strain on healthcare systems, particularly in resource‐limited settings [1, 2, 3, 4]. CVDs remain the leading cause of mortality worldwide, with a disproportionate burden in low‐ and middle‐income countries (LMICs) [5, 6]. Between 2008 and 2017, high‐income countries produced 81.1% of global CVD research but accounted for just 8.1% of CVD‐related disability‐adjusted life‐year (DALY) losses and 8.5% of deaths. In contrast, LMICs, where the burden of CVD is highest, generated only 2.8% of research despite accounting for 59.5% and 57.1% of global CVD DALY losses and death rates, respectively [7].

Observational studies have reported a bidirectional association between major depressive disorder (MDD) and CAD, with depression linked to increased risk of cardiac events, and CAD associated with higher prevalence of depressive symptoms [4, 8, 9, 10, 11]. For example, a study by Lichtman and colleagues [12, 13] found that depression prevalence among CAD patients ranges from 15% to 30%, nearly two to three times higher than in the general population, whereas a separate study reported that patients with major depression faced nearly double the risk of cardiac mortality over a 5‐year period. Despite increasing recognition of this association, much of the evidence comes from European (EUR) ancestry populations, with limited data from LMICs and other underrepresented groups [14, 15, 16]. In many LMICs, depression remains underdiagnosed and undertreated due to stigma, insufficient mental health infrastructure and limited access to care [17, 18]. As a result, the presence and directionality of the association between CAD and MDD in these populations remain unclear.

Mendelian randomisation (MR) provides a robust framework for reducing biases from confounding and reverse causation, providing insights into the biological mechanisms underlying disease risk [16, 19, 20, 21, 22]. Although recent MR studies have investigated the bidirectional relationship between depression and CVDs, findings consistently suggest that genetically predicted depression increases the risk of CAD, whereas the reverse direction—from CAD to depression—has shown null or inconclusive results [23, 24, 25]. However, these analyses have been conducted predominantly in populations of EUR ancestry, limiting the generalisability of their findings. Given the known differences in genetic architecture and environmental exposures, expanding MR research to include diverse populations is critical to ensure that genetic insights translate into meaningful public health interventions globally [23, 26, 27].

This study employs a bidirectional two‐sample MR approach to investigate the causal relationship between MDD and CAD across multiple ancestry groups. By identifying population‐specific genetic determinants of MDD and CAD, this work aims to improve our understanding of the biological mechanisms underlying these conditions in diverse populations and contribute to more inclusive genetic research.

## 2 Methods

### 2.1 MR Methodology

There are three core instrumental variable (IV) assumptions which need to be held true in an MR study to ensure valid causal inference: (1) the genetic variant must be strongly associated with the exposure of interest; (2) the genetic variant must not be associated with any confounders that influence both the exposure and outcome and (3) the genetic variant must influence the outcome solely through the exposure, without any alternative pathways [28, 29, 30]. Violations of these assumptions can bias effect estimates and limit their interpretability [31].

**FIGURE 1:**
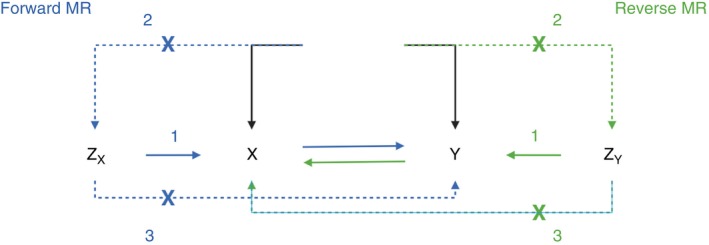
Core assumptions of a bidirectional Mendelian randomisation (MR) analysis. The genetic variants (*Z_x_* and *Zᵧ*) serve as IVs and must satisfy three key assumptions: (1) Relevance: *Z_x_* is associated with exposure *X*, and *Zᵧ* is associated with exposure *Y*. (2) Independence: The genetic variants are not associated with confounders (U). (3) Exclusion restriction: The genetic variants influence the outcome (*Y* for forward MR, *X* for reverse MR) only through their respective exposure.

In this bidirectional two‐sample MR study, we assess the causal relationship between MDD and CAD in both directions. First, the forward MR analysis tests whether genetic liability to MDD influences the risk of developing CAD. Then, the reverse MR analysis examines whether genetic predisposition to CAD affects the likelihood of developing MDD. To achieve this, we used genome‐wide association study (GWAS) summary statistics from large, ancestry‐specific cohorts to identify independent genetic variants robustly associated with each trait. We then applied MR methods, including inverse‐variance weighted (IVW) analysis and sensitivity analyses such as MR‐Egger and weighted median approaches, to assess causal effects while accounting for potential violations of IV assumptions. The relationship between these assumptions and the structure of our MR analyses is illustrated in Figure 1.

### 2.2 Study Design

**FIGURE 2:**
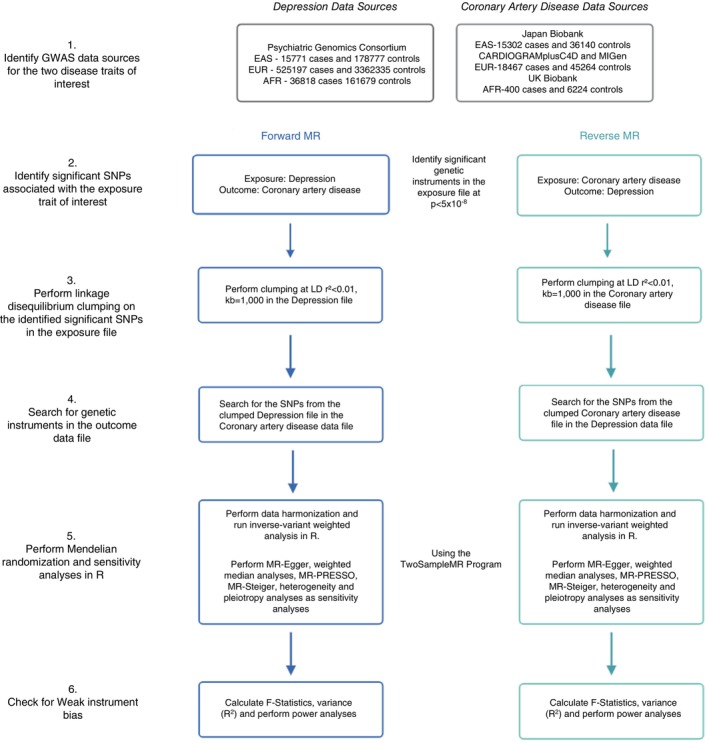
Study workflow for bidirectional two‐sample Mendelian randomisation (MR) analysis of major depressive disorder (MDD) and coronary artery disease (CAD). The workflow outlines the bidirectional two‐sample MR approach used to investigate the association between MDD and CAD in East Asian (EAS), EUR and African (AFR) populations.

The analytical framework for this study is depicted in Figure 2. IVs were selected based on the three core MR assumptions outlined earlier. In the forward MR (MDD‐to‐CAD), IVs were selected from a GWAS of MDD, while in the reverse MR (CAD‐to‐MDD), IVs were selected from a GWAS of CAD. A genome‐wide significance threshold of *p* < 5 × 10^−8^ was applied to select IVs for the exposures; however, when fewer than 10 variants remained after clumping, suggestive thresholds of *p* < 5 × 10^−7^ and *p* < 5 × 10^−6^ were instead applied.

Clumping was performed (*r* ^2^ < 0.01, 500 kb window) to retain only independent IVs and avoid bias due to linkage disequilibrium (LD), where nearby genetic variants are inherited together due to their physical proximity on the genome. This step ensures that the selected IVs represent an independent single nucleotide polymorphism (SNP) rather than correlated variants, which reduces bias and overestimation of causal effects [32].

After selecting SNPs associated with the exposure, we identified these variants in the GWAS of the respective outcome traits—CAD for the forward MR and MDD for the reverse MR—to extract their corresponding allele frequencies and effect estimates. This ensured that the same genetic variants were assessed in both datasets, allowing for a valid two‐sample MR analysis. In cases where an IV was absent from the outcome GWAS, a proxy SNP in high LD (*r* > 0.8) was identified using ancestry‐specific genotype data from Phase 3 Version 5 of the 1000 Genomes Project. Proxies were determined using the online tool SNiPA, ensuring that ancestry specificity was maintained across all analyses [33].

### 2.3 Data Sources

Genetic instruments for MDD were sourced from ancestry‐stratified GWAS conducted by the Psychiatric Genomics Consortium (PGC). Depression was defined based on a combination of self‐reports, structured clinical interviews, symptom‐based and medical health records. Ancestry‐specific GWAS included individuals of AFR, EAS, South Asian and EUR descent. For this analysis, summary statistics included the PGC multi‐ancestry GWAS of MDD, which incorporated individuals of AFR ancestry, a targeted GWAS of depression in EAS populations, and a large‐scale EUR GWAS that expanded upon previous multi‐ancestry findings [24, 25, 27].

Summary statistics for CAD were obtained from the GWAS Catalog and UK Biobank. CAD was defined based on medical records and clinical diagnoses within hospital‐based registries and research cohorts. Because of this, the specific exposure phenotype derived by each GWAS was broadly defined, with CAD, ischemic heart disease, myocardial infarction and angina all included within the respective GWAS. Sources included the Coronary Artery Disease Genome‐Wide Replication and Meta‐Analysis plus the Coronary Artery Disease Consortium, Japan Biobank and UK Biobank [34, 35, 36]. The summary information of each selected data source is provided in Supporting Information S1.

To ensure the independence of exposure and outcome data sources within ancestry groups, as required by the two‐sample MR framework, we undertook a review of the study cohorts contributing to both the MDD and CAD GWAS. Published methodologies, consortium documentation and supplementary materials were examined to identify any potential sample overlap. Based on this review, no sample overlap was identified for AFR, EAS or EUR populations; for the latter, outcome data excluding UK Biobank participants was used to avoid duplication.

### 2.4 Statistical Analysis

After identifying significant SNPs for each trait, we harmonised the effect alleles to ensure consistency in the direction of effect estimates across both exposure and outcome datasets. The primary bidirectional MR analysis was performed using the IVW approach under a fixed‐effects model, which provides the most precise causal estimate when all IVs are valid. Forward (MDD‐to‐CAD) and reverse (CAD‐to‐MDD) MR analyses were conducted separately. As both MDD and CAD are treated as binary traits in the underlying genetic studies, effect estimates were reported as odds ratios (ORs), and no unit transformation was required. All analyses were conducted in R (version 4.3.2) using the TwoSampleMR (0.6.8) and MR (0.10.0) packages [37, 38, 39, 40].

To assess the robustness of our findings and evaluate potential violations of MR assumptions, we performed a series of sensitivity analyses (Supporting Information [Link], [Link], [Link], [Link], [Link], [Link], [Link]). The MR‐Steiger directionality test was used to confirm whether the selected genetic instruments explained more variance in the exposure than in the outcome, supporting the assumption that the exposure precedes the outcome. A failure in the MR‐Steiger test suggests possible misspecification of causality, where the instruments may be more strongly associated with the outcome than the exposure [38, 41].

To address potential violations of the exclusion restriction assumption, which states that genetic variants should only influence the outcome through the exposure, we applied MR‐Egger regression and the weighted median estimator. These methods help mitigate the impact of pleiotropy, a key concern in MR studies where genetic variants may affect the outcome through alternative biological pathways. MR‐Egger accounts for directional pleiotropy by allowing for a non‐zero intercept, while the weighted median provides robust causal estimates even if up to 50% of the instruments are invalid [41, 42, 43]. We also implemented the MR‐PRESSO method to detect and correct for pleiotropic outliers, reducing the risk that observed effects were driven by invalid instruments [44].

We further assessed the strength of the genetic instruments using the *F*‐statistic, which evaluates the strength of association between each SNP and the exposure. *F*‐statistics greater than 10 were considered indicative of sufficiently strong instruments, reducing the likelihood of weak instrument bias that could attenuate results towards the null [45]. To evaluate the consistency of causal estimates across individual SNPs, we assessed heterogeneity using Cochran's *Q* statistic and *I* ^2^ statistics. Diagnostic tools were also applied, including forest plots, funnel plots and leave‐one‐out analyses, to visually inspect the influence of individual SNPs on the overall effect estimate.

Finally, we conducted statistical power calculations based on the available sample sizes for each trait. We estimated the minimum detectable ORs for each direction of the MR analyses at 80% power, across a range of plausible values representing the proportion of variance in the exposure explained by the genetic instruments (*R* ^2^); an 80% power threshold was considered sufficient to detect potentially meaningful effects [40]. This approach accounts for the uncertainty inherent in power estimation for MR studies, where assumptions about true effect size and instrument strength can vary, and offers a more transparent alternative to static a priori or post hoc power estimates.

To account for multiple comparisons in the bidirectional analysis, we applied a Bonferroni correction to control for the risk of type I error.

### 2.5 Ethical Approval

All GWAS datasets used in this study have obtained ethical approval and informed consent from participants. As this study exclusively utilised publicly available summary‐level data, no additional ethical approval was required.

## 3 Results

### 3.1 Bidirectional Two‐Sample MR Analysis

**FIGURE 3:**
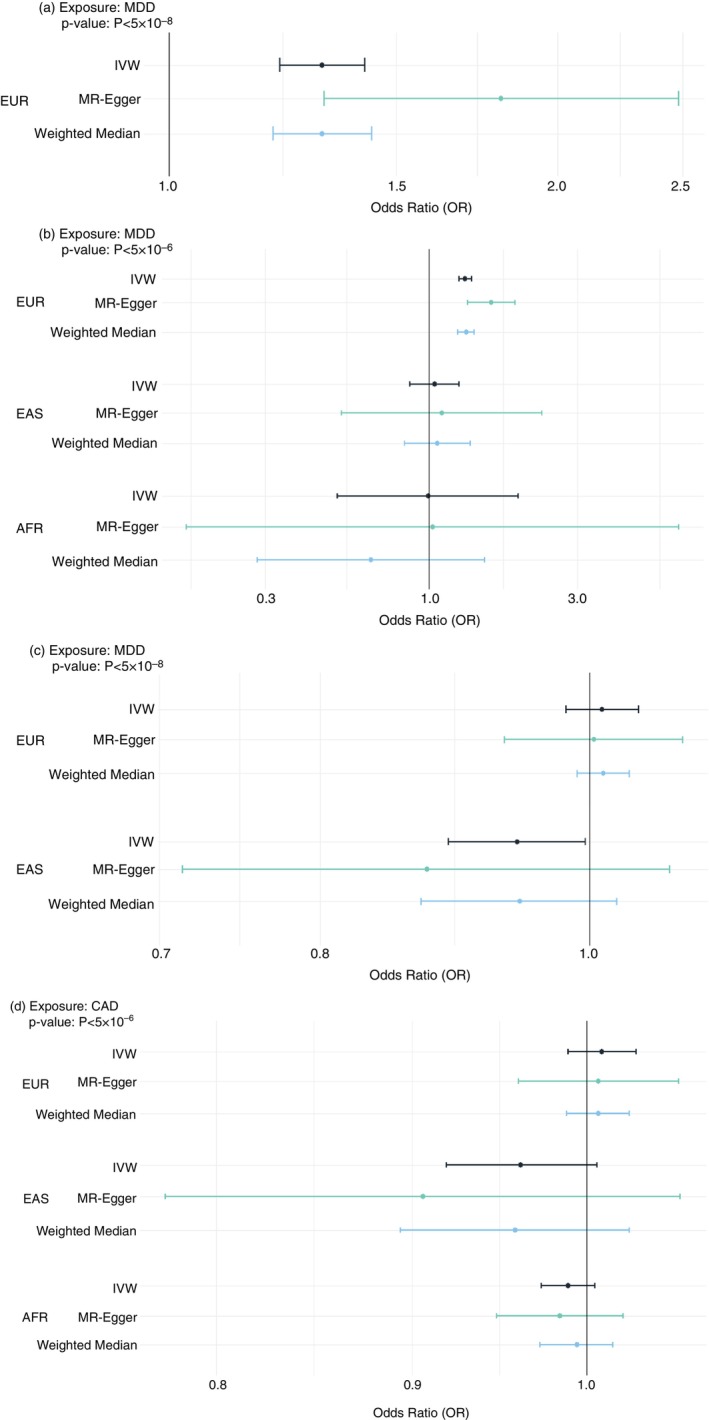
Bidirectional two‐sample Mendelian randomisation estimates for major depressive disorder (MDD) and coronary artery disease (CAD) across diverse ancestry populations. Causal effects of MDD on CAD in East Asian (EAS), European (EUR) and African (AFR) populations are shown at (a) *p* < 5 × 10^−8^ and (b) *p* < 5 × 10^−6^ thresholds. Causal effects of CAD on MDD in EAS, EUR and AFR populations are shown at (c) *p* < 5 × 10^−8^ and (d) *p* < 5 × 10^−6^ thresholds. Results are presented as odds ratios (ORs) using the inverse‐variance weighted (IVW), MR‐Egger and weighted median methods.

MR analysis in EURs showed evidence of a causal effect of MDD on CAD, where a genetically predicted increase in liability to MDD was associated with 31% higher odds of CAD (IVW OR = 1.314; 95% CI: 1.218–1.419; *p* < 0.001). In the reverse direction, no association was observed between genetically predicted liability to CAD and risk of MDD (IVW OR = 1.011; 95% CI: 0.981–1.042; *p* = 0.477), and findings remained consistent when more relaxed *p* value thresholds were applied (Figure 3 and Supporting Information S3).

In EASs, the IVW estimate suggested a potential inverse association between CAD and MDD (IVW OR = 0.942; 95% CI: 0.890–0.997; *p* = 0.040). However, this association was not supported by alternative MR methods or sensitivity analyses and may represent a false‐positive finding, as it did not remain statistically significant after Bonferroni correction for multiple testing across the bidirectional analyses (adjusted significance threshold: *p* < 0.0125). Moreover, the association was not observed when more relaxed *p* value thresholds were applied for instrument selection (IVW OR = 0.961; 95% CI: 0.919–1.006; *p* = 0.091). No evidence of a causal effect was detected in the reverse direction in EASs, nor in either direction in AFR populations (Figure 3).

### 3.2 Statistical Power and Heterogeneity

We estimated the minimum detectable ORs at 80% power across a range of plausible instrument strengths (*R* ^2^ = 0.01–0.3), using outcome sample sizes for each ancestry group. These power calculations were not based on the observed MR results but were instead conducted across various *R* ^2^ values to assess the sensitivity of our study under different instrument strengths (Supporting Information S7 and S8).

Power curves showed that, for a given *R* ^2^, the detectable OR at 80% power varied across populations due to differences in sample size. For instance, at *R* ^2^ = 0.05, the minimum detectable ORs in the forward MR were approximately 1.12 in EURs, 1.13 in EASs and 1.70 in AFRs. In the reverse direction, the corresponding ORs were approximately 1.02 in EURs, 1.11 in EASs and 1.08 in AFRs. At a higher instrument strength (e.g., *R* ^2^ = 0.1), the minimum detectable ORs in the forward MR decreased to 1.08 in EURs, 1.09 in EASs and 1.50 in AFRs, whereas in the reverse MR, the values were 1.08 in EURs, 1.02 in EASs and 1.06 in AFRs. These findings illustrate the lower sensitivity to modest effect sizes in smaller samples, particularly for AFR ancestry populations.

To evaluate the strength of the IVs, we calculated the median *F*‐statistics for each ancestry group using the *R* ^2^ values derived from the individual SNPs in our analysis (Supporting Information S6). In the forward MR, with MDD as the exposure, EUR instruments were consistently strong (*F* ≥ 10), with a median *F*‐statistic of 15 at *p* < 5 × 10^−8^. In contrast, instruments for EAS and AFR populations were weak (*F* < 10) across all *p* value thresholds.

In the reverse MR, with CAD as the exposure, both EUR and EAS instruments were strong, with a median *F*‐statistic of 15 for EASs at *p* < 5 × 10^−8^. As with the forward MR, the median *F*‐statistics for AFR populations remained weak, regardless of the *p* value threshold.

Cochran's *Q* tests were used to evaluate heterogeneity among the IVs for each ancestry group at the genome‐wide significance threshold of *p* < 5 × 10^−8^. In the forward MR, with MDD as the exposure, significant heterogeneity was observed among EUR instruments using both IVW (*Q* = 352.92, df = 197, *p* < 0.001) and MR‐Egger methods (*Q* = 345.63, df = 196, *p* < 0.001). Heterogeneity could not be assessed in EASs due to an insufficient number of variants, and no estimates were available for AFR ancestry populations due to the lack of instruments. In the reverse MR analysis, with CAD as the exposure, significant heterogeneity was again observed in EURs (IVW *Q* = 213.44, df = 44, *p* = 0.000; MR‐Egger *Q* = 213.23, df = 43, *p* = 0.000), while no evidence of heterogeneity was found in EASs (IVW *Q* = 16.49, df = 17, *p* = 0.490; MR‐Egger *Q* = 15.91, df = 16, *p* = 0.459). Heterogeneity statistics for AFR ancestry populations could not be calculated due to the lack of available instruments. Full heterogeneity results at both *p* < 5 × 10^−8^ and *p* < 5 × 10^−6^ thresholds are provided in Supporting Information S4.

### 3.3 Sensitivity Analyses

To assess the robustness of our MR results and check for any violations of key assumptions, we performed several sensitivity analyses (Table 1 and Supporting Information [Link], [Link], [Link], [Link], [Link], [Link], [Link]). The MR‐Steiger test indicated a high failure rate in the forward direction (MDD as the exposure) for EUR populations (54% at *p* < 5 × 10^−8^; 60% at *p* < 5 × 10^−6^), suggesting that many instruments were more strongly associated with CAD than MDD. Failure rates were lower in AFRs (47%) and EASs (0%), indicating some population differences in instrument validity. In the reverse MR direction (CAD as the exposure), all instruments passed the MR‐Steiger test across each of the populations.

**TABLE 1:** Sensitivity analyses for bidirectional two‐sample Mendelian randomisation (MR) analyses of major depressive disorder (MDD) and coronary artery disease (CAD) across East Asian (EAS), European (EUR) and African (AFR) populations.

Ancestry	*p* threshold	Cochrane *Q* test (IVW)			MR‐Egger intercept test			Median *F*‐statistic	MR‐Steiger (number of invalid instruments)
		*Q*	*Q*_df	*p*	Intercept	SE	*p*		
Forward MR (exposure: depression)									
EAS	*p* < 5 × 10^−8^	Insufficient number of IVs			Insufficient number of IVs			NA	NA
	*p* < 5 × 10^−6^	5.00	6	0.540	−0.005	0.033	0.883	9	0
EUR	*p* < 5 × 10^−8^	352.92	197	< 0.001	−0.008	0.004	0.042	15	107
	*p* < 5 × 10^−6^	848.82	575	< 0.001	−0.004	0.002	0.022	10	345
AFR	*p* < 5 × 10^−8^	No available IVs			No available IVs			NA	NA
	*p* < 5 × 10^−6^	23.1	18	0.19	−0.003	0.079	0.968	4	9
Reverse MR (exposure: coronary artery disease)									
EAS	*p* < 5 × 10^−8^	16.49	17	0.490	0.012	0.015	0.448	15	0
	*p* < 5 × 10^−6^	37.67	38	0.490	0.008	0.010	0.428	10	0
EUR	*p* < 5 × 10^−8^	213.44	44	< 0.001	0.001	0.003	0.841	16	0
	*p* < 5 × 10^−6^	328.10	98	< 0.001	0.000	0.020	0.929	10	0
AFR	*p* < 5 × 10^−8^	No available IVs			No available IVs			NA	0
	*p* < 5 × 10^−6^	7.99	15	0.920	0.003	0.012	0.766	2	0

*Note*: Results include Cochran's *Q* test for heterogeneity, MR‐Egger intercept test for directional pleiotropy, median *F*‐statistics for instrument strength and the number of invalid instruments identified by the MR‐Steiger test at significance thresholds of *p* < 5 × 10^−8^ and *p* < 5 × 10^−6^.

We compared IVW estimates with MR‐Egger and weighted median models to evaluate consistency across methods. In EURs, MR‐Egger regression suggested directional pleiotropy at both thresholds (intercepts: 0.992 and 0.996, both *p* < 0.001), and MR‐PRESSO detected significant global pleiotropy (*p* < 2 × 10^−4^) in all forward and reverse MR analyses. No pleiotropy was detected in EAS or AFR populations.

MR‐PRESSO identified significant global pleiotropy in both the forward and reverse analyses among EURs (*p* < 0.0002), with several outlier SNPs detected in each direction. In the forward analysis, correction for three outliers yielded a substantially stronger effect estimate (OR = 0.29, *p* = 9.28 × 10^−14^), although the distortion test was not significant (*p* = 0.738), suggesting the correction did not materially alter the interpretation. In the reverse analysis, five outliers were removed, but the corrected estimate was negligible (OR = 2.83 × 10^−4^, *p* = 0.974), although the distortion was significant (*p* = 0.007), indicating a marked influence of outliers on the original estimate. No outliers were identified in EASs, and tests could not be conducted in AFRs due to insufficient instruments.

## 4 Discussion

This study presents a large‐scale bidirectional two‐sample MR analysis to evaluate the causal relationship between MDD and CAD across EAS, EUR and AFR populations. Our results indicate a causal effect of genetically predicted MDD on increased CAD risk in EURs, aligning with previous MR findings. In the reverse direction, we identified a weak inverse association between CAD and MDD in EASs, with borderline significance (*p* = 0.040) in the primary IVW analysis. This suggests that while depression may increase the risk of heart disease in EUR populations, there is also preliminary evidence indicating that genetic liability to CAD might be associated with a slightly reduced risk of depression in EASs. However, as this finding did not meet the Bonferroni‐adjusted threshold, the results from this analysis require replication and further validation.

The observed causal effect of MDD on CAD among EURs aligns with both MR and epidemiological evidence linking depression to elevated cardiovascular risk [14, 46]. Biological pathways such as systemic inflammation, hypothalamic–pituitary–adrenal axis dysregulation, and metabolic disruption are proposed mediators in this relationship [47, 48, 49, 50]. Behavioral factors, including physical inactivity, poor dietary habits and smoking, which are more prevalent among individuals with depression, likely also contribute to increased CAD susceptibility. Taken together, these mechanisms highlight how depression may influence CAD risk through both physiological and behavioral pathways, underscoring the importance of integrating mental health into cardiovascular risk reduction frameworks.

In addition to confirming established risk pathways, this study also identified a novel potential association between genetic liability to CAD and a reduced risk of MDD in EAS populations. The IVW analysis suggested a modest inverse association between CAD and MDD risk; however, this effect was close to the threshold for statistical significance and not supported by sensitivity analyses. Specifically, alternative MR methods such as the weighted median and MR‐Egger produced non‐significant estimates with wide confidence intervals. Although no outliers were identified by MR‐PRESSO and instrument strength was adequate, albeit in the borderline range (*F* = 15), these findings raise uncertainty about the robustness of the result.

One possible explanation for the inverse association is subtle horizontal pleiotropy, where genetic variants independently influence both CAD and MDD through distinct pathways, potentially in opposing directions, thereby violating the MR assumption that genetic instruments affect the outcome only through the exposure. Although MR‐Egger did not detect significant directional pleiotropy, the borderline IVW estimate may still reflect unbalanced pleiotropic effects—such as genetic variants influencing intermediate traits like metabolic risk factors—which are not fully captured by standard sensitivity analyses.

Additionally, it remains possible that the instruments used do not fully capture the biological pathways linking CAD and MDD in EAS ancestry individuals. Phenotypic heterogeneity, differences in sample characteristics, and environmental variation across cohorts, including population‐specific gene–environment interactions, may contribute to the observed effect [51]. In EAS populations, differences in environmental exposures or lifestyle factors may modify the relationship between genetic liability to CAD and depression risk. In addition, variation in how depression is phenotypically defined across studies may complicate interpretation. Psychiatric symptoms are often underreported in some cultural contexts, potentially distorting genetic correlations, and clinical criteria for MDD diagnosis may differ across settings [52, 53]. As a result, the depression phenotype captured in EAS GWAS may not be directly comparable to that in EUR cohorts, introducing additional heterogeneity into cross‐population MR analyses.

Although novel, the inverse association observed is inconsistent with the null results reported in earlier MR studies, underscoring the need for replication using larger, well‐powered datasets and pleiotropy‐robust methods. Moreover, as this association did not meet the Bonferroni‐corrected significance threshold, these findings should be considered preliminary and warrant further study to clarify whether this signal reflects true biological effects or methodological artefacts [14, 15, 54, 55, 56, 57, 58, 59].

More broadly, while MR is designed to mitigate confounding and reverse causation, the complex, multifactorial nature of both MDD and CAD necessitates interpretation of causal findings alongside broader epidemiological and clinical evidence. Shared biological pathways, such as systemic inflammation or physical inactivity, may contribute to both conditions, and psychiatric symptoms may only emerge clinically following a CAD event [60, 61, 62]. Conversely, undiagnosed depression may precede CAD onset but only come to medical attention during cardiovascular assessment [63, 64]. Although such diagnostic patterns are unlikely to bias MR estimates, they remain important for understanding how these conditions present and progress. Although MR offers valuable insight into underlying causal pathways, incorporating both genetic and non‐genetic evidence is essential to fully capture ancestry‐specific variation and inform equitable, population‐relevant public health strategies.

### 4.1 Methodological Considerations and Limitations

Several methodological considerations warrant attention. First, genetic instrument selection for EAS and AFR populations relied on less stringent *p* value thresholds and clumping parameters due to the limited availability of genome‐wide significant SNPs, especially in the forward MR direction (MDD as the exposure). Although this approach was necessary to increase power and enable bidirectional analyses, it may have introduced weak instrument bias, reduced the precision of causal estimates, and increased sensitivity to specific analytical choices, potentially attenuating true effects. Relaxing instrument selection criteria can also increase the risk of including variants with weaker associations to the exposure, which may bias MR estimates towards the null. This is especially relevant given that the resulting *F*‐statistics for several analyses fell within borderline ranges, raising additional concerns about the strength of the instruments and the robustness of the causal inference.

Second, although we performed multiple sensitivity analyses, residual pleiotropy remains a concern. In the EUR dataset, MR‐Egger intercept tests and MR‐PRESSO analyses indicated horizontal pleiotropy, suggesting that some genetic variants may influence outcomes through pathways unrelated to the exposure. In EASs, while the inverse association between CAD and MDD reached borderline significance in the primary IVW analysis, neither MR‐Egger nor weighted median methods produced significant estimates, and confidence intervals were wide. This inconsistency suggests that the result may be vulnerable to pleiotropic bias and highlights the need for cautious interpretation.

Selection bias is another important limitation to consider. The GWAS datasets used in this study were derived from large biobank cohorts, many of which include volunteers who tend to be healthier and are less likely to have severe mental or physical health conditions compared to the general population [65, 66]. This may have biased effect estimates if the genetic determinants of depression and CAD differ between biobank participants and the general population. Recruitment strategies, healthcare access and socio‐economic disparities are also likely to influence the demographics of individuals included in the respective datasets.

Finally, statistical power varied across populations. Although the EUR and EAS GWAS provided well‐powered data for MR, the AFR CAD datasets were underpowered, limiting instrument strength and the interpretability of findings in that population. This highlights the need for larger, ancestrally diverse GWAS to enable more robust and generalisable multi‐ancestry MR analyses.

### 4.2 Strengths and Future Directions

By incorporating data from EAS, EUR and AFR populations, this study broadens the scope of existing research by incorporating diverse populations and proposing new hypotheses, including the potential causal relationship between CAD and MDD in EAS populations. The use of multiple MR methods (IVW, MR‐Egger and weighted median) and pleiotropy‐robust sensitivity analyses strengthens confidence in the primary forward MR findings in EURs, while also highlighting methodological uncertainties where the results differed from those in EAS populations.

Future studies should prioritise expanding GWAS in non‐EUR populations, as larger, ancestrally diverse samples will strengthen instruments, reduce bias and enable more robust and interpretable MR analyses. Incorporating polygenic risk scores, gene–environment interaction models and multi‐omics approaches—such as transcriptomics, methylation or metabolomics—will further clarify the biological pathways linking depression and CAD. Additionally, replicating and reassessing the inverse association between CAD and MDD in EAS populations is crucial to evaluate its robustness and biological validity. Such research will be critical for developing tailored, ancestry‐informed prevention strategies that bridge mental and cardiovascular health.

## 5 Conclusion

This study provides evidence for a causal relationship between MDD and CAD, emphasising the need to consider psychological factors in CVD prevention and treatment. Although a preliminary inverse association between CAD and MDD was observed in EAS populations, this finding did not meet corrected significance thresholds and was not supported by sensitivity analyses, suggesting that residual confounding or methodological biases may have influenced the result and that replication is required. Collectively, these results highlight the need for greater ancestral diversity in genetic research to ensure that causal inferences are robust and globally relevant, ultimately supporting more equitable public health strategies.

## Conflicts of Interest

The authors declare no conflicts of interest.

## Supporting information


**Data S1:** tmi70051‐sup‐0001‐supinfo.xlsx.

**Data S2:** tmi70051‐sup‐0002‐supinfo.xlsx.

**Data S3:** tmi70051‐sup‐0003‐supinfo.xlsx.

**Data S4:** tmi70051‐sup‐0004‐supinfo.xlsx.

**Data S5:** tmi70051‐sup‐0005‐supinfo.xlsx.

**Data S6:** tmi70051‐sup‐0006‐supinfo.xlsx.

**Data S7:** tmi70051‐sup‐0007‐supinfo.xlsx.

**Data S8:** tmi70051‐sup‐0008‐supinfo.pdf.

**Data S9:** tmi70051‐sup‐0009‐supinfo.xlsx.

**Data S10:** tmi70051‐sup‐0010‐supinfo.pdf.

**Data S11:** tmi70051‐sup‐0011‐supinfo.pdf.

## Data Availability

The genome‐wide association summary statistics data used in this study are publicly available at https://www.ebi.ac.uk/gwas/downloads/summary‐statistics and https://pgc.unc.edu/for‐researchers/download‐results/. All software programs used in this paper are listed and referenced in the Methods. The full coding script used to perform the analysis is available from the corresponding authors upon request.
