# Supplementary material for: Causal Relationships Between Major Depressive Disorder and Coronary Artery Disease Across Diverse Populations: A Bidirectional Mendelian Randomisation Study
Source: Trop Med Int Health. 2025 Oct 15;31(1):80–90. doi: 10.1111/tmi.70051 (PMC12775895; doi:10.1111/tmi.70051)
Supplement: Supplementary file 8 — Data S8: tmi70051‐sup‐0008‐supinfo.pdf. [file TMI-31-80-s006.pdf]

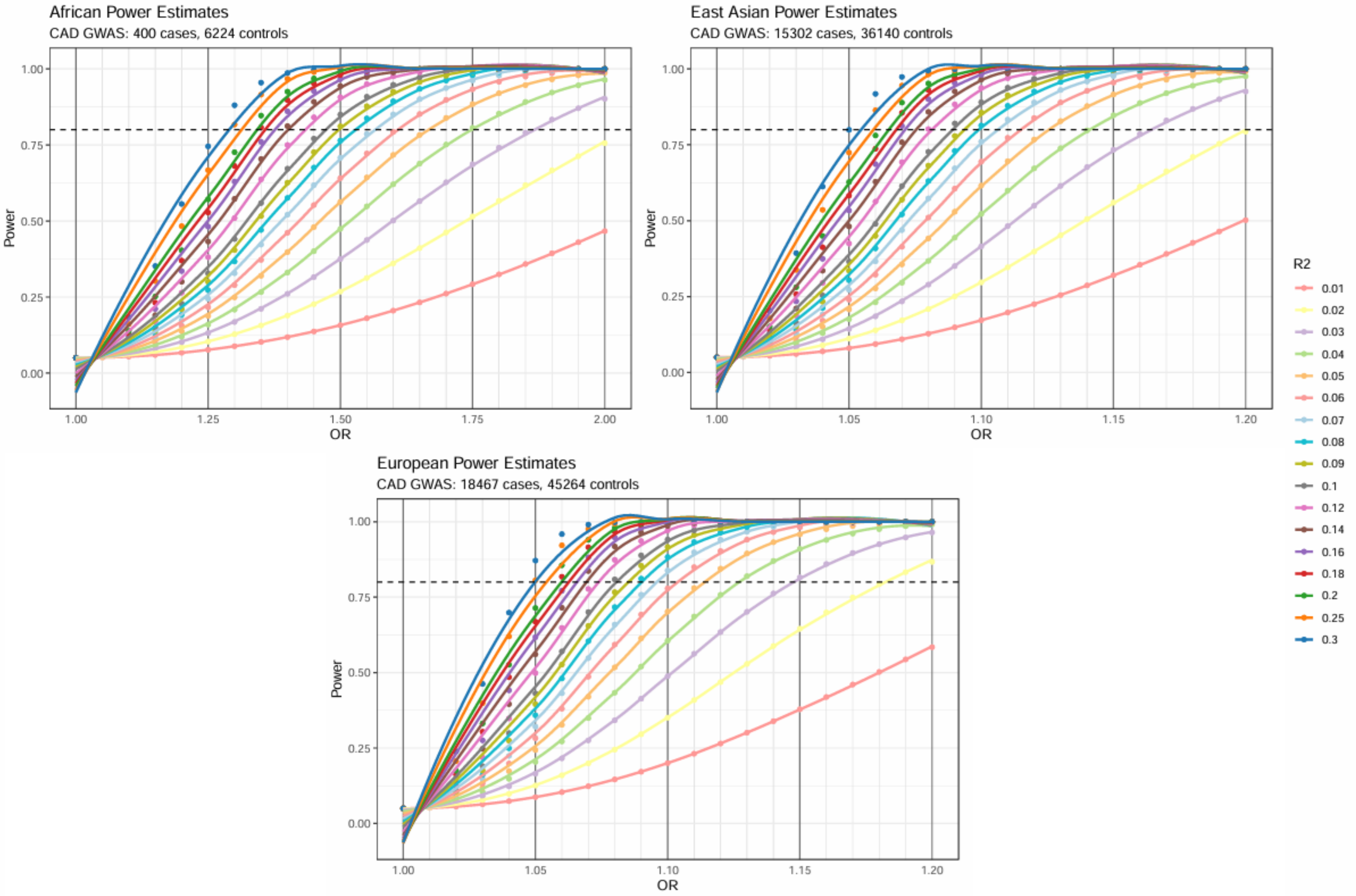

African Power Estimates
CAD GWAS: 400 cases, 6224 controls
Power
1.00
0.75
0.50
0.25
0.00
1.00
1.25
1.50
1.75
2.00
OR
East Asian Power Estimates
CAD GWAS: 15302 cases, 36140 controls
Power
1.00
0.75
0.50
0.25
0.00
1.00
1.05
1.10
1.15
1.20
OR
European Power Estimates
CAD GWAS: 18467 cases, 45264 controls
Power
1.00
0.75
0.50
0.25
0.00
1.00
1.05
1.10
1.15
1.20
OR
R2
0.01
0.02
0.03
0.04
0.05
0.06
0.07
0.08
0.09
0.1
0.12
0.14
0.16
0.18
0.2
0.25
0.3

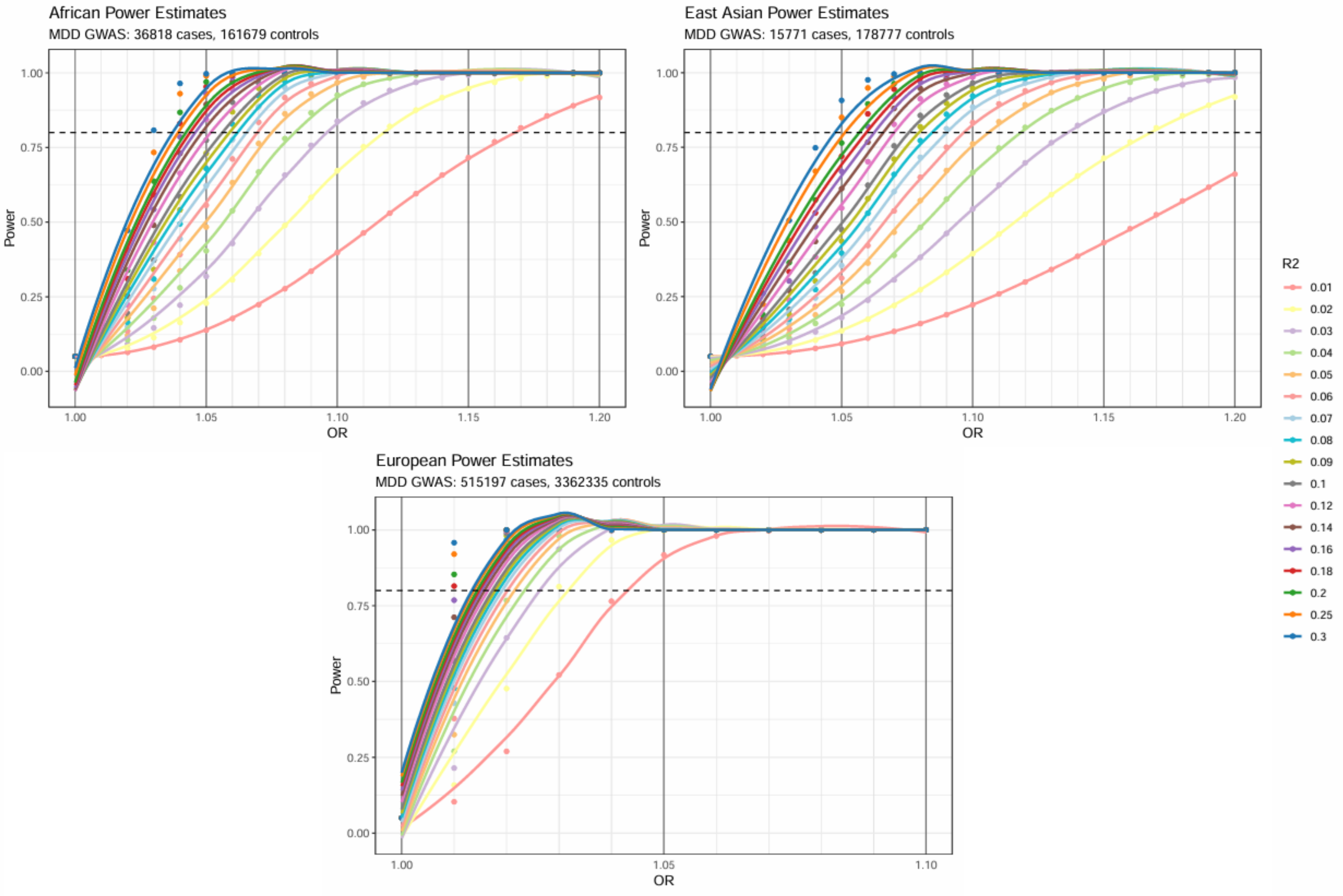
African Power Estimates
MDD GWAS: 36818 cases, 161679 controls
East Asian Power Estimates
MDD GWAS: 15771 cases, 178777 controls
European Power Estimates
MDD GWAS: 515197 cases, 3362335 controls
Power
OR
R2
0.01
0.02
0.03
0.04
0.05
0.06
0.07
0.08
0.09
0.1
0.12
0.14
0.16
0.18
0.2
0.25
0.3
