# Supplementary material for: Causal Relationships Between Major Depressive Disorder and Coronary Artery Disease Across Diverse Populations: A Bidirectional Mendelian Randomisation Study
Source: Trop Med Int Health. 2025 Oct 15;31(1):80–90. doi: 10.1111/tmi.70051 (PMC12775895; doi:10.1111/tmi.70051)

Depression MR Plots (10^{-6})

EAS

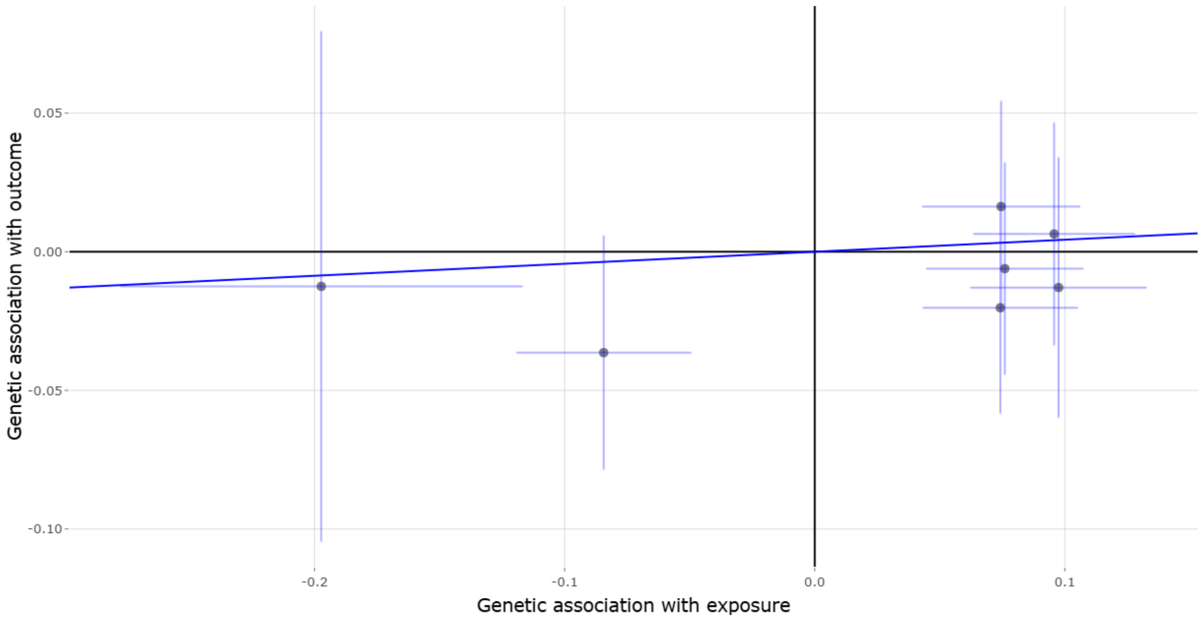

EUR

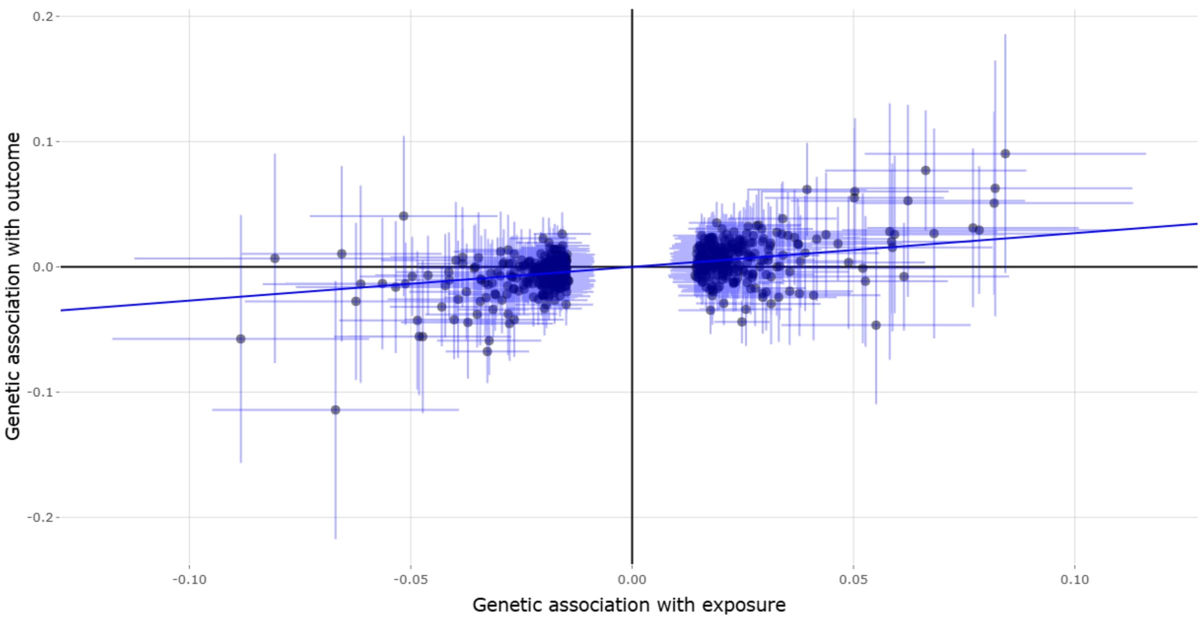

AFR

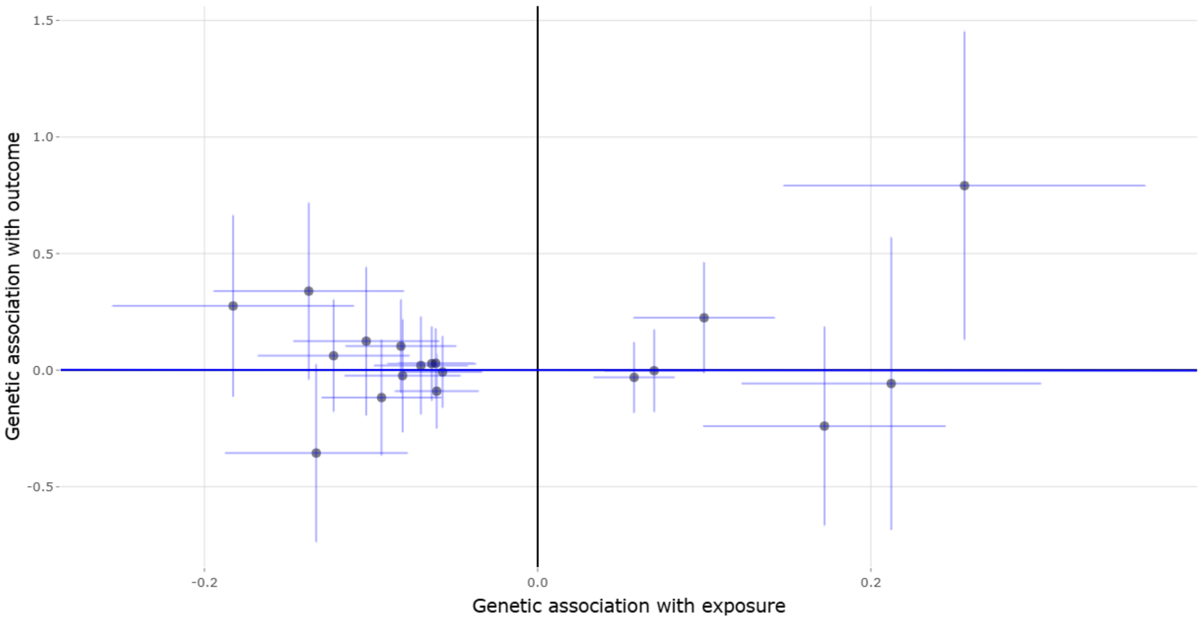

Depression MR Plots (10^{-8})

EUR

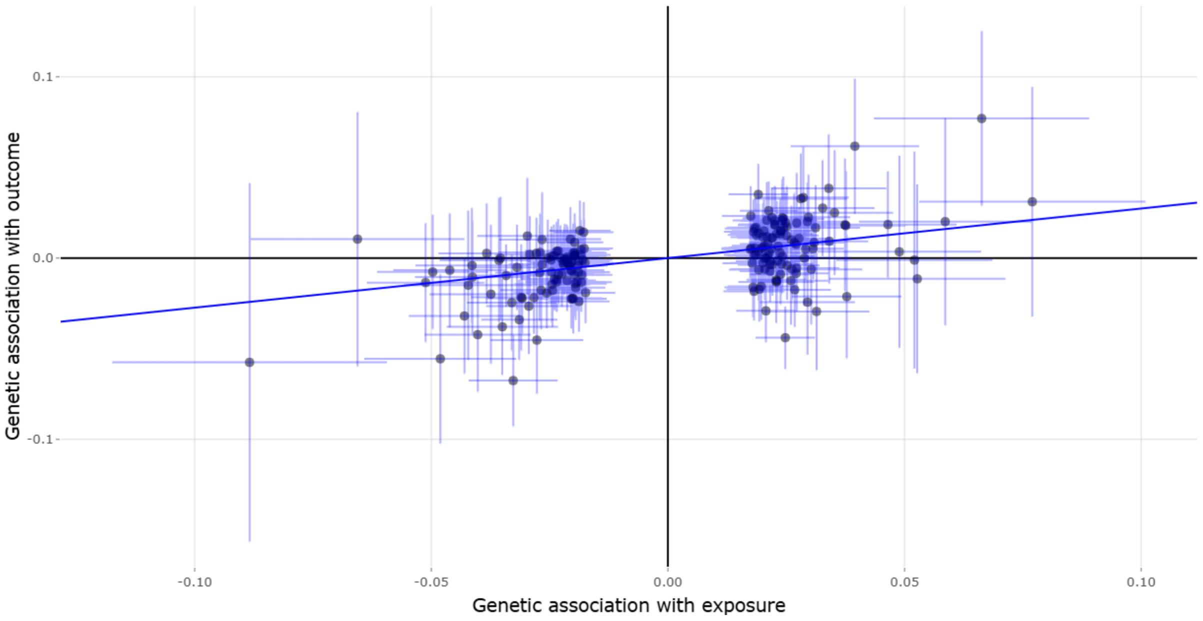

Depression Leave-one-out Analysis (10^{-6})

EAS

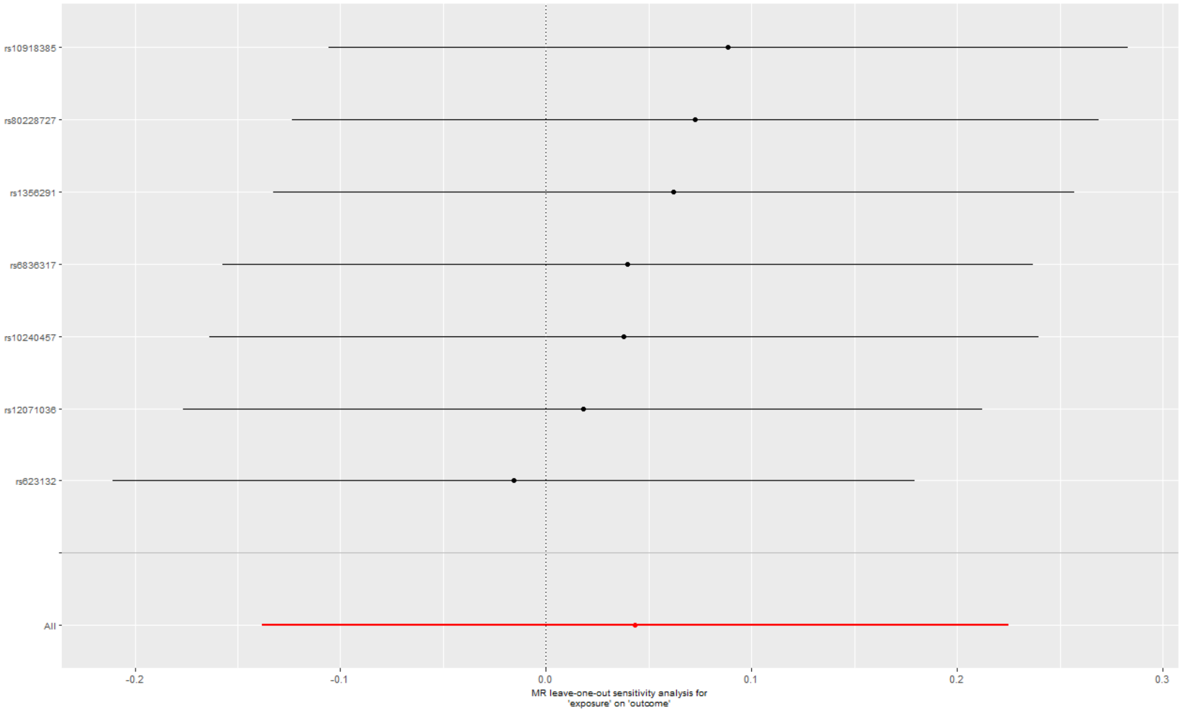

EUR

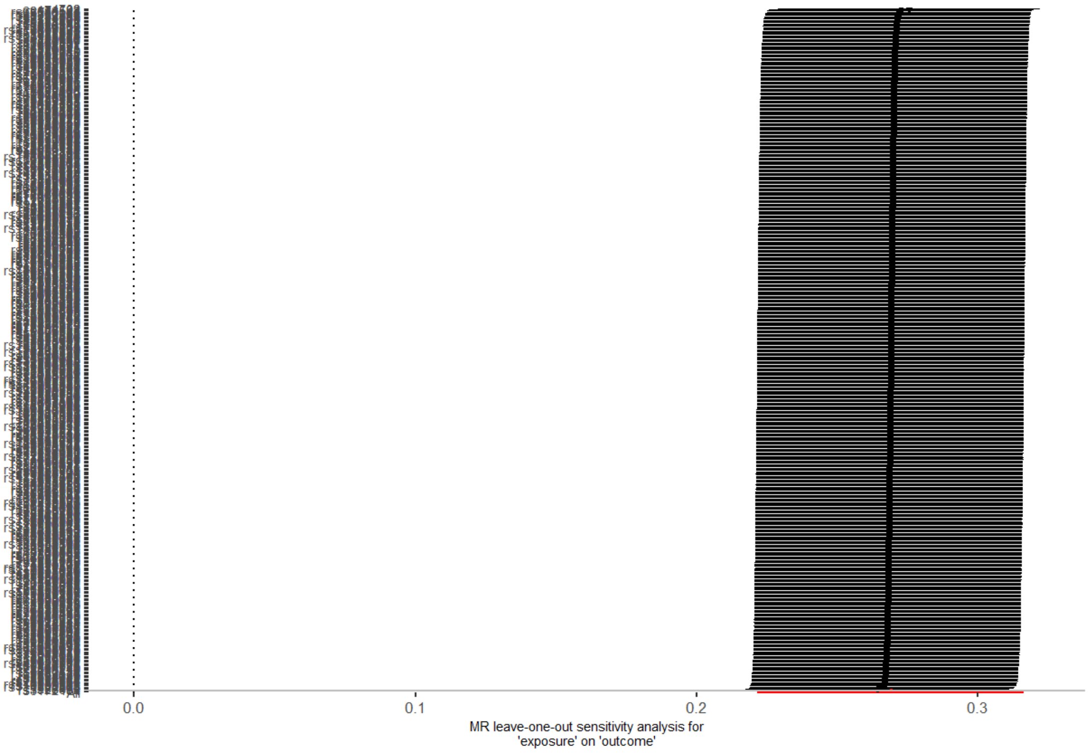

AFR

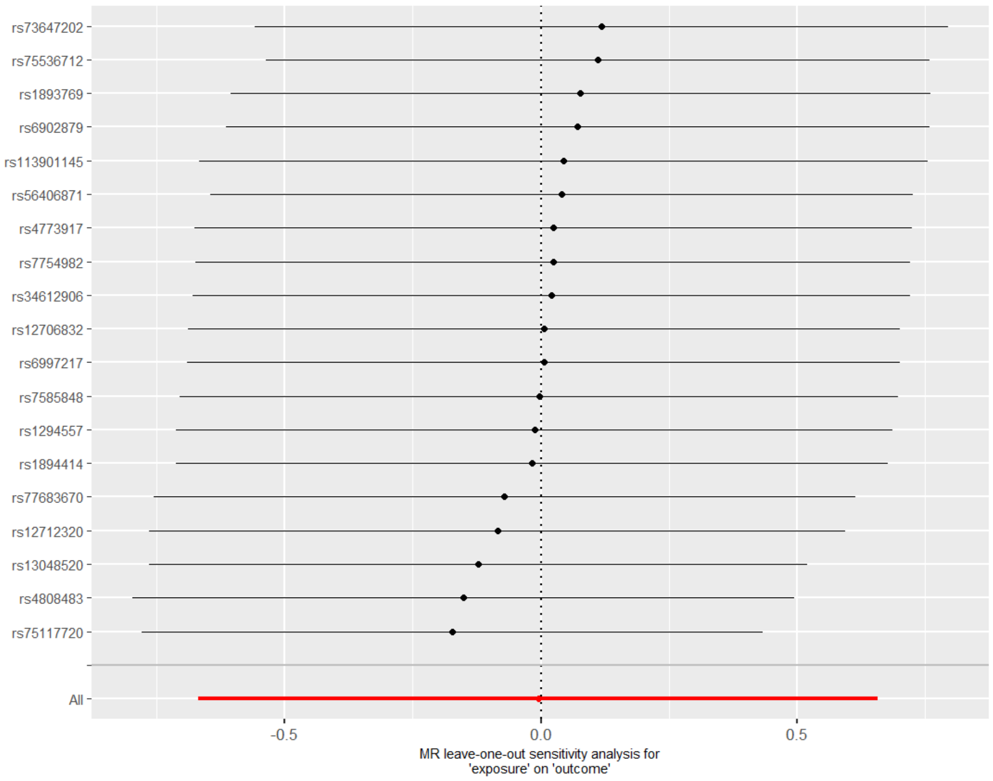

Depression Leave-one-out Analysis (10^{-8})

EUR

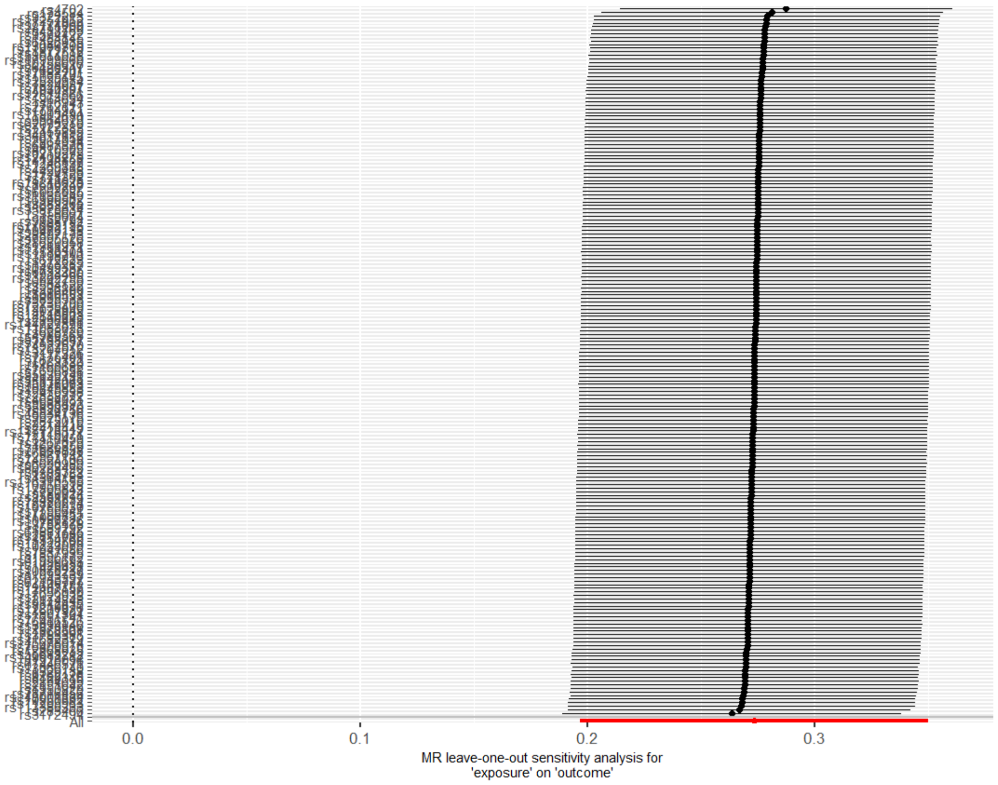

Depression Forest Plots (10^{-6})

EAS

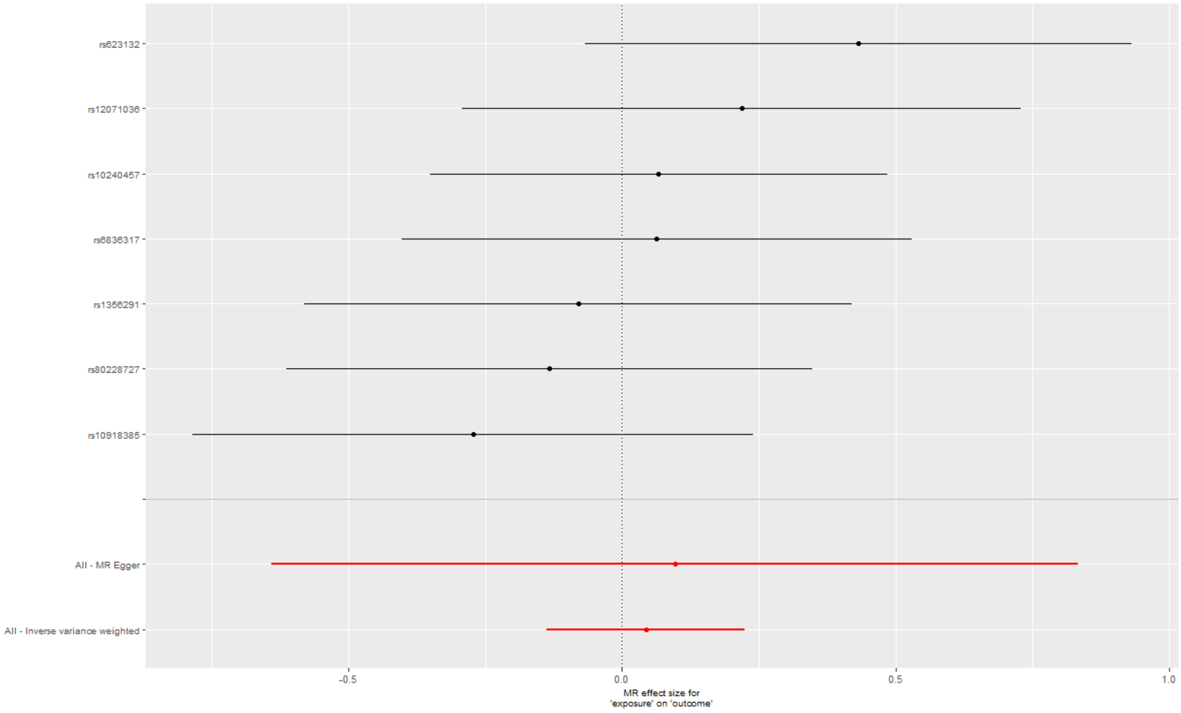

EUR

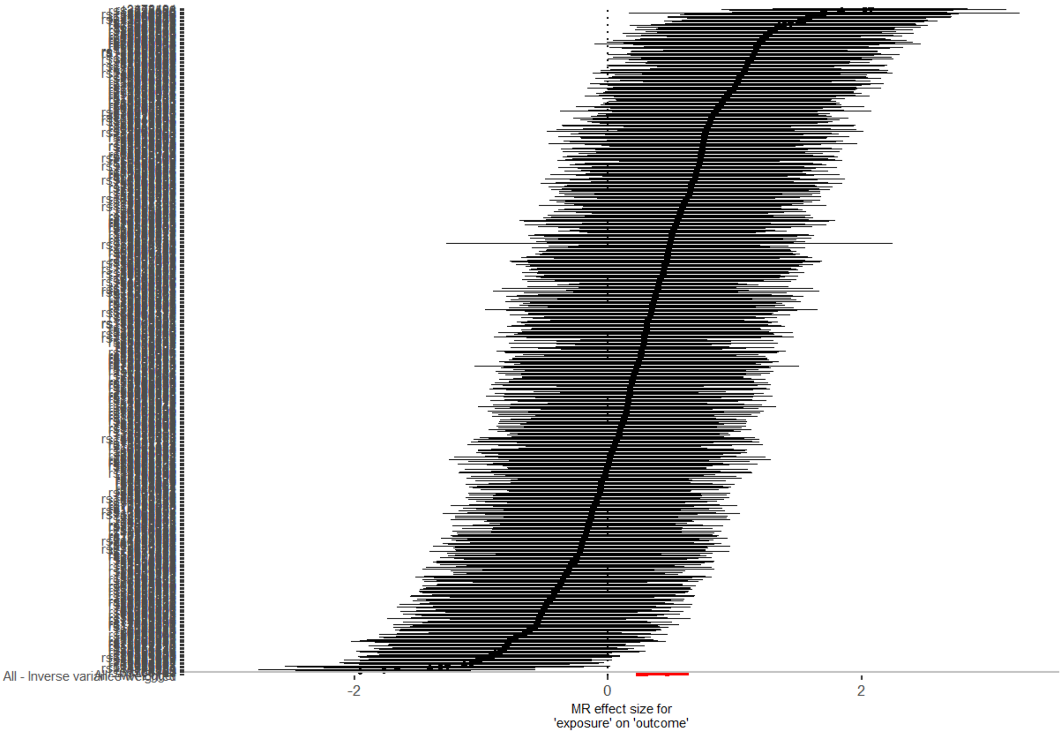

AFR

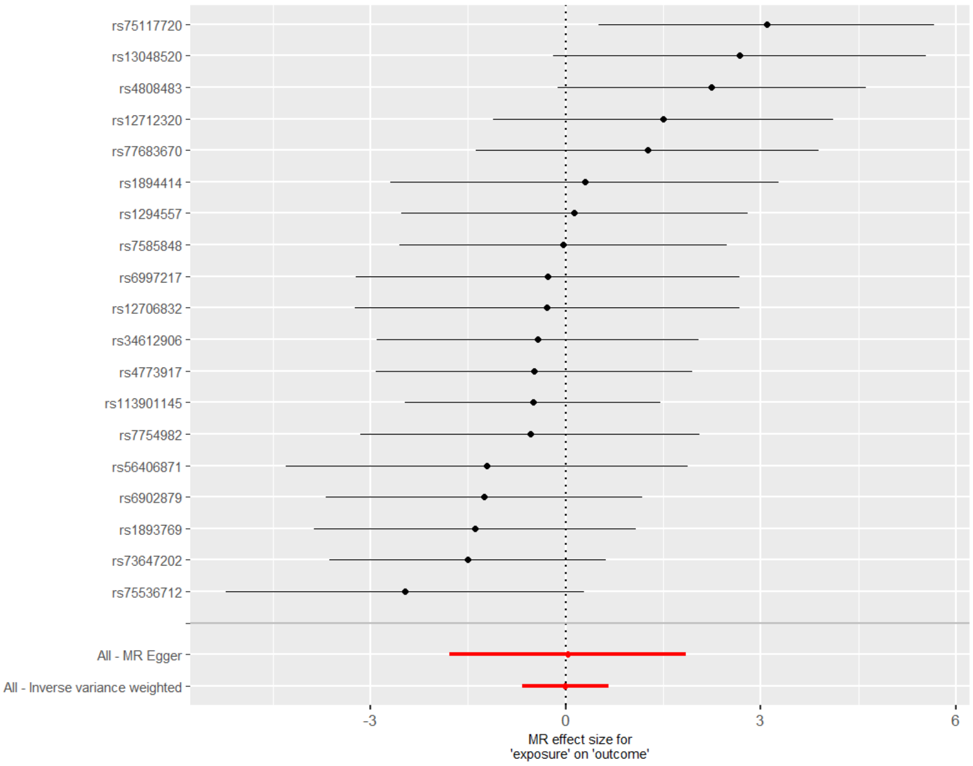

Depression Forest Plots (10^{-8})

EUR

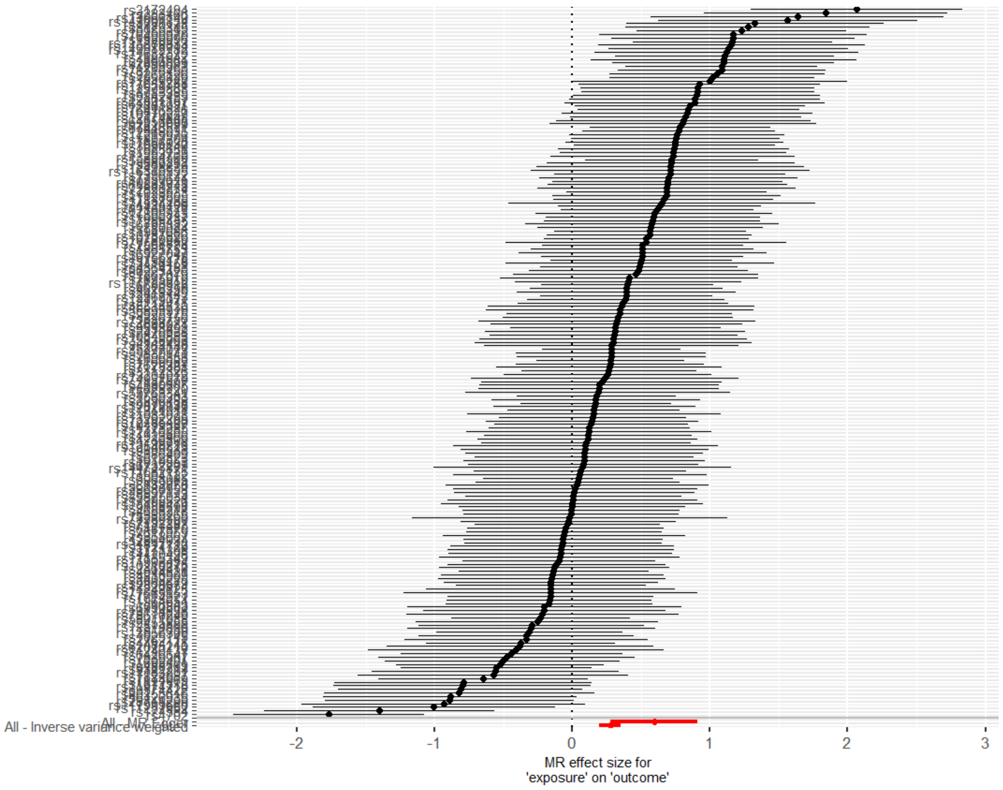

Depression Funnel Plots (10^{-6})

EAS

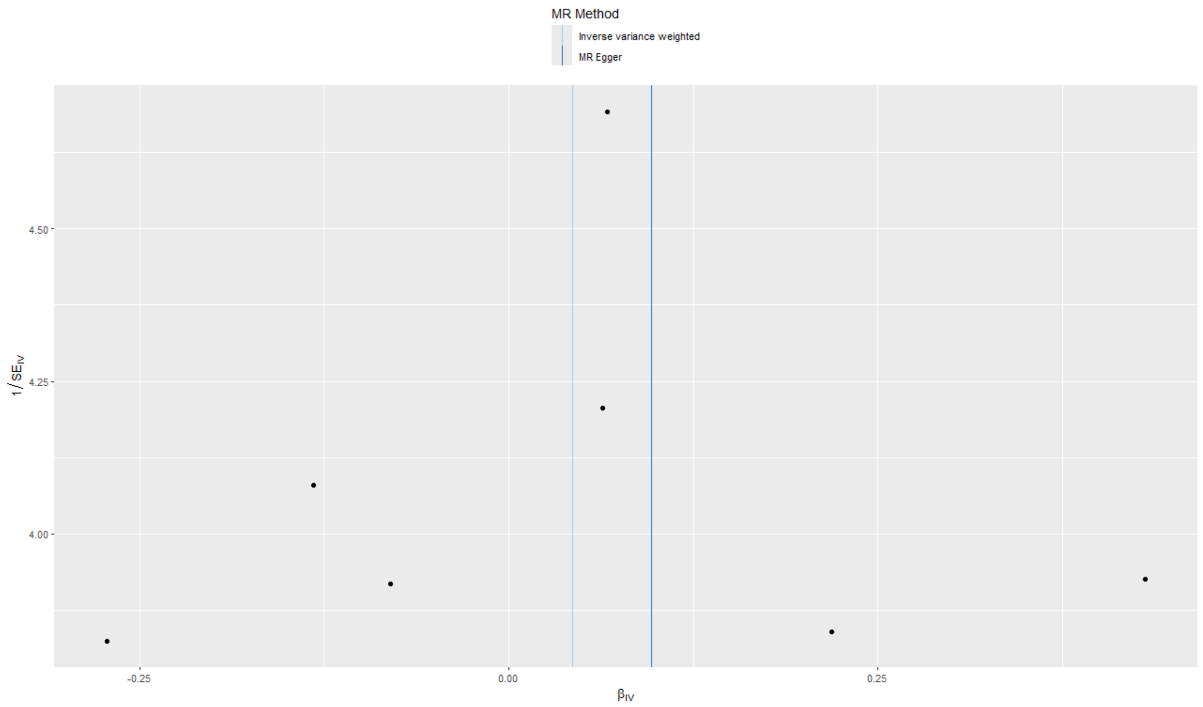

EUR

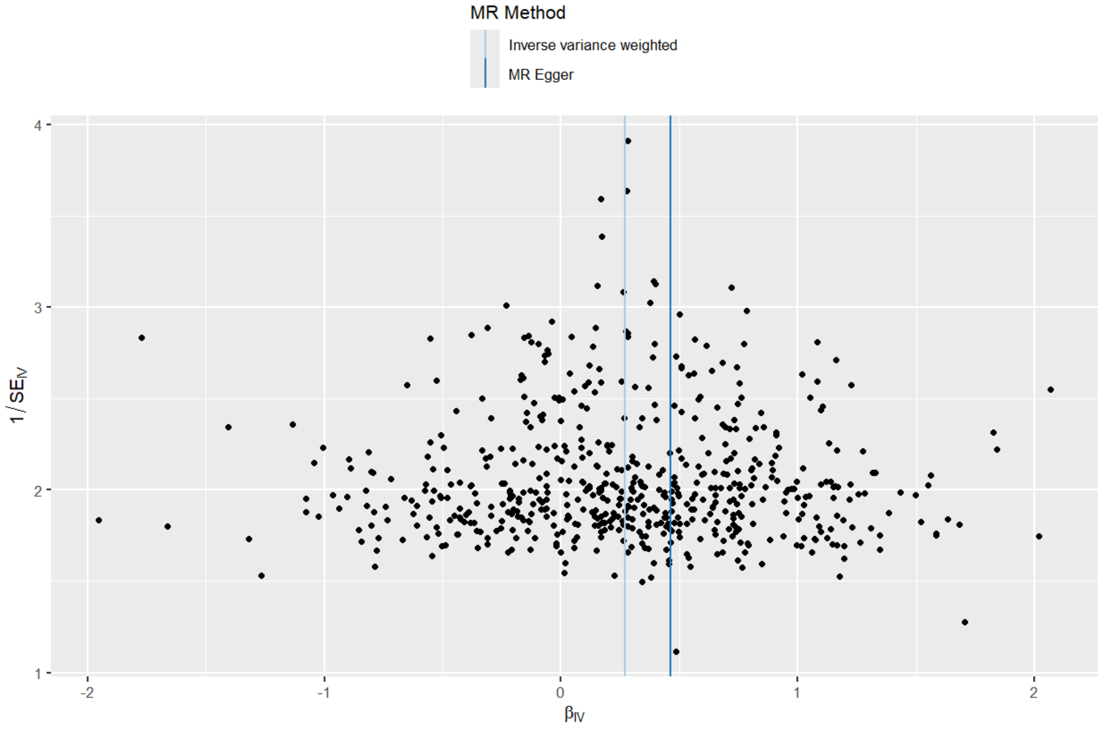

AFR

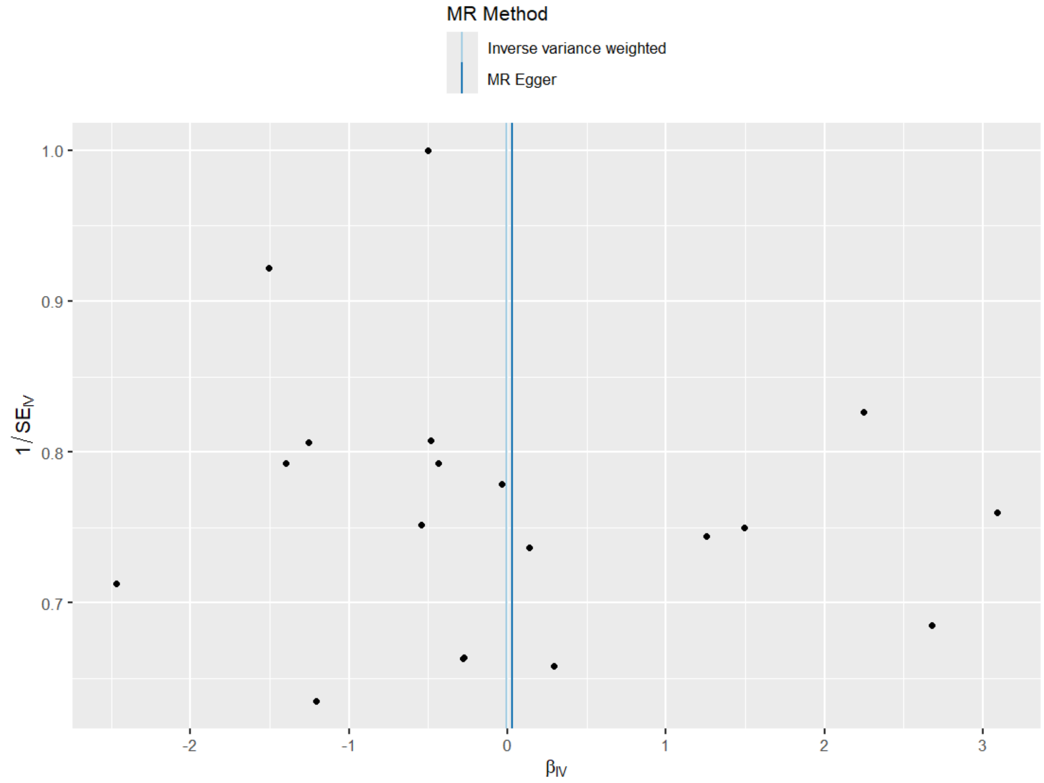

Depression Funnel Plots (10^{-8})

EUR

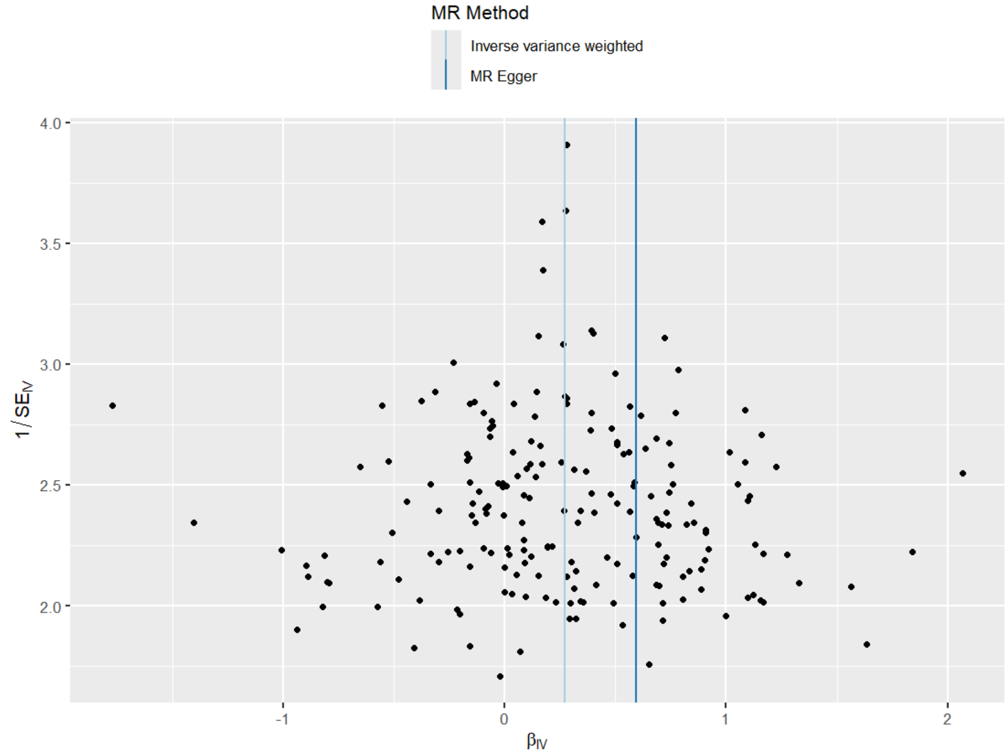

CAD MR Plots (10^{-6})

EAS

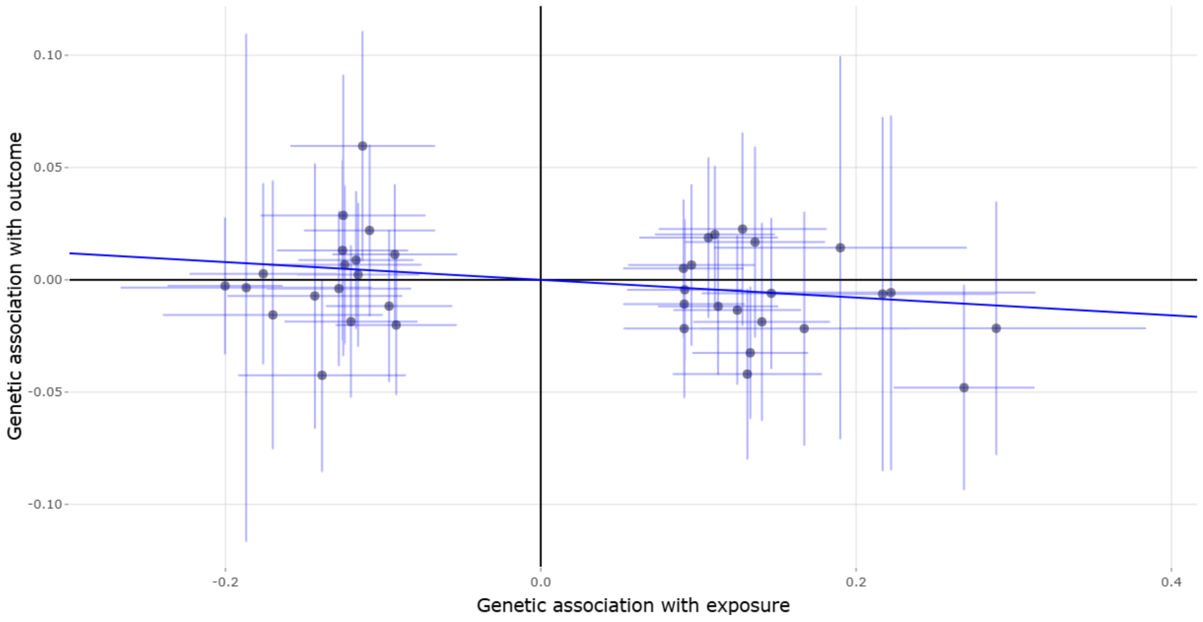

EUR

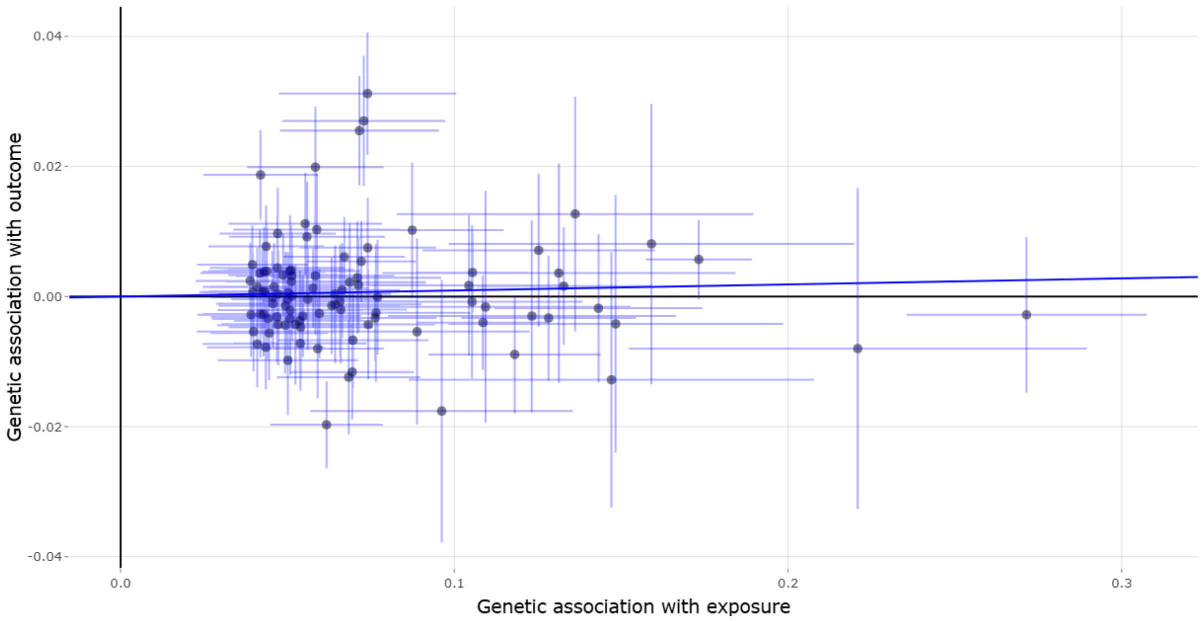

AFR

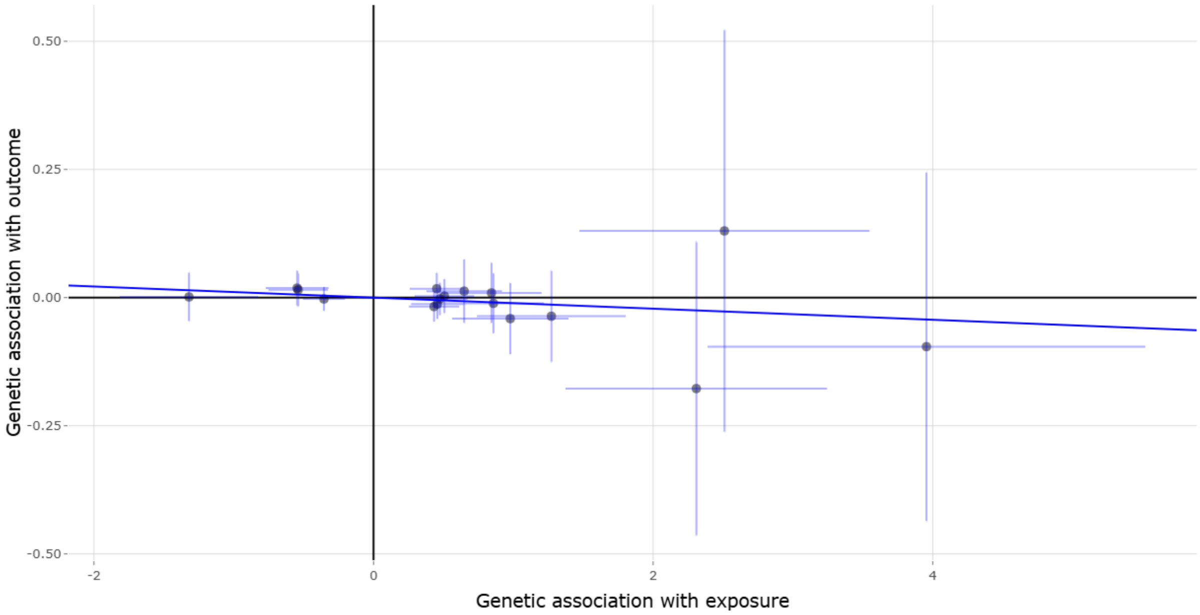

CAD MR Plots (10^{-8})

EAS

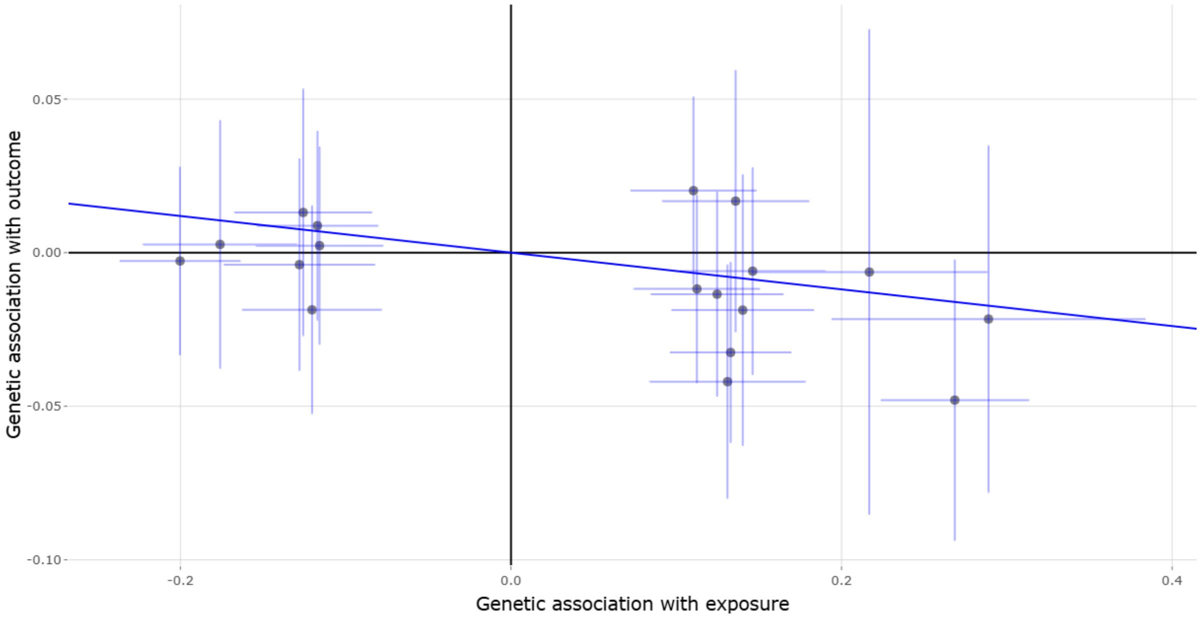

EUR

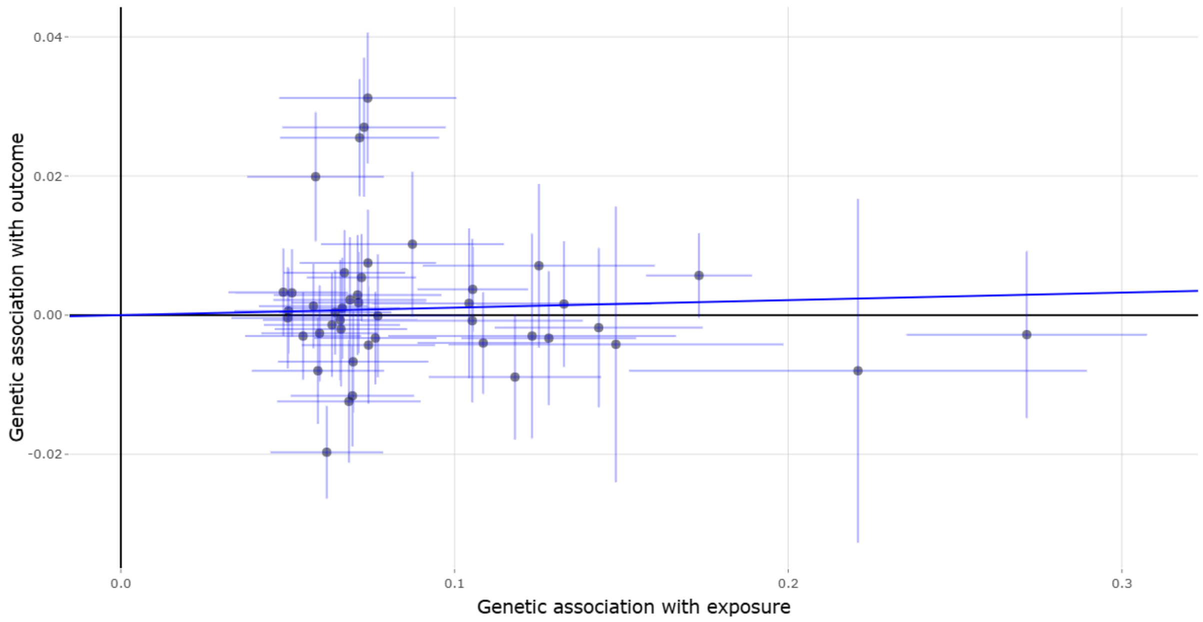

CAD Leave-one-out Analysis (10^{-6})

EAS

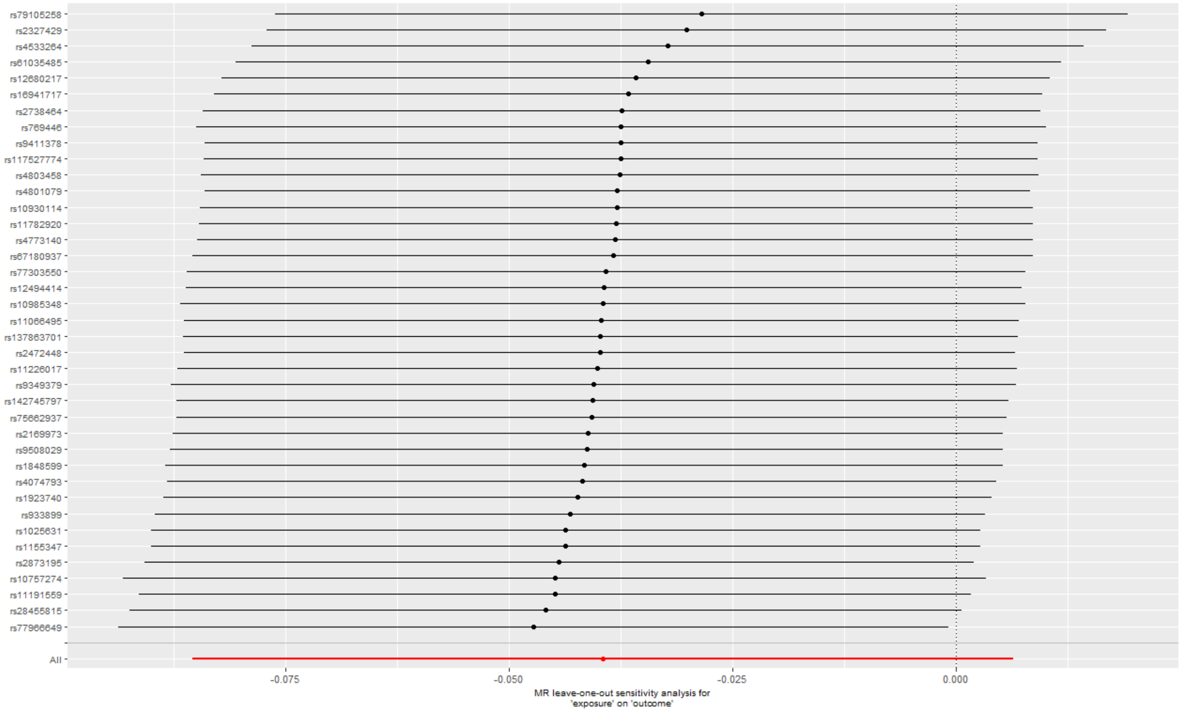

EUR

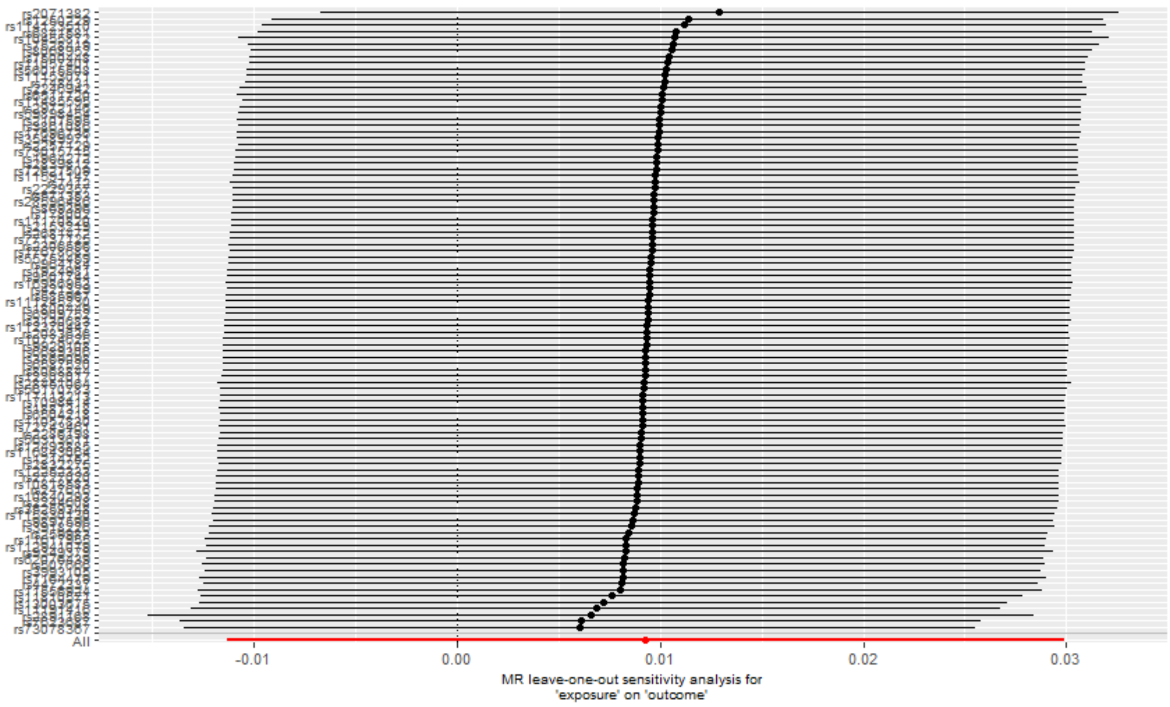

rs2071382
All
-0.01
0.00
0.01
0.02
0.03
MR leave-one-out sensitivity analysis for
'exposure' on 'outcome'

AFR

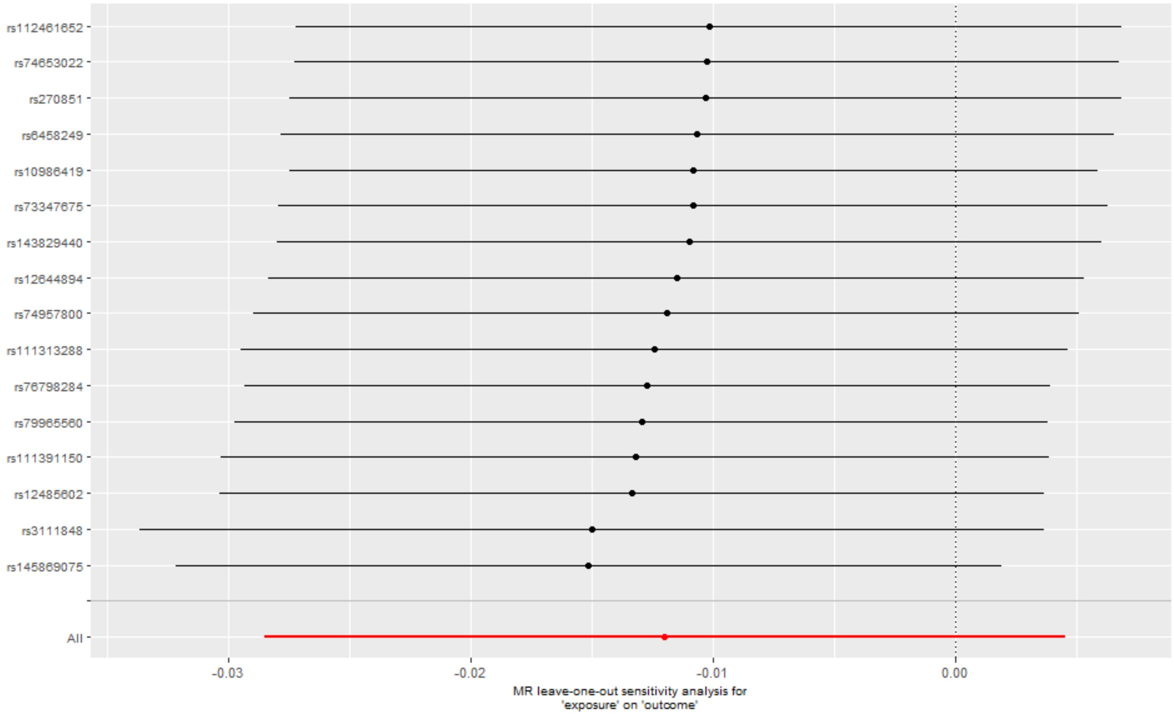

rs112461652
rs74653022
rs270851
rs6458249
rs10986419
rs73347675
rs143829440
rs12644894
rs74957800
rs111313288
rs76798284
rs79965560
rs111391150
rs12485602
rs3111848
rs145869075
All
-0.03
-0.02
-0.01
0.00
MR leave-one-out sensitivity analysis for
'exposure' on 'outcome'

CAD Leave-one-out Analysis (10^{-8})

EAS

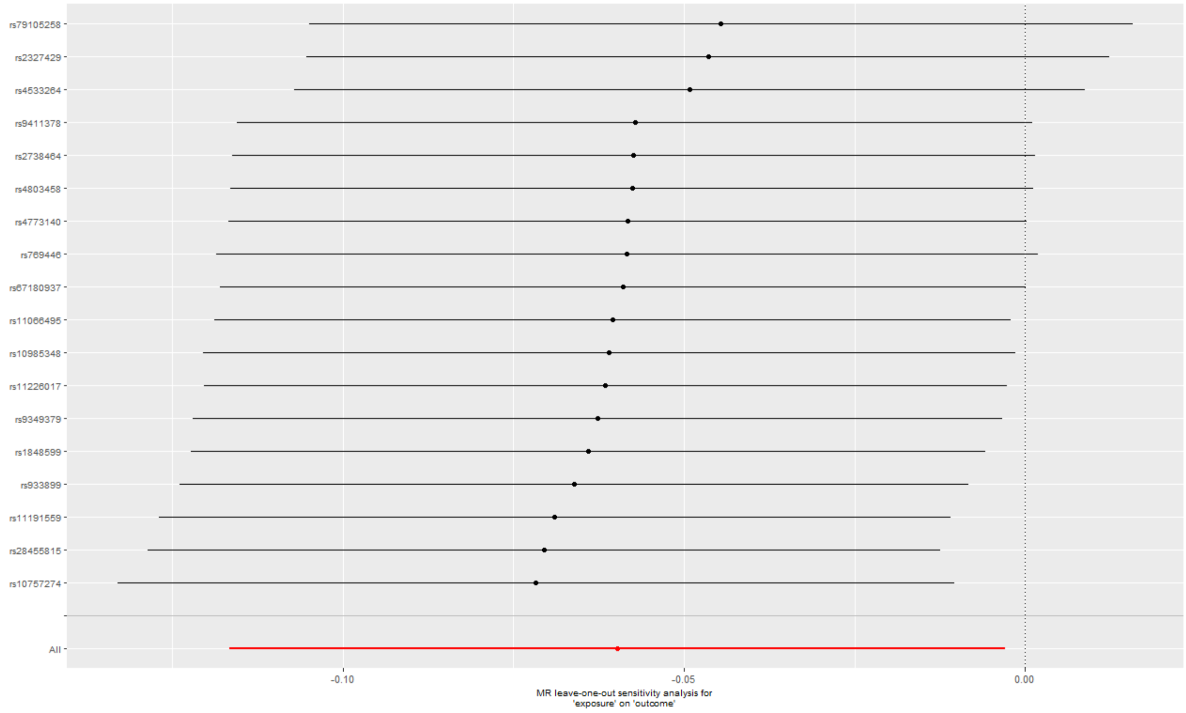

EUR

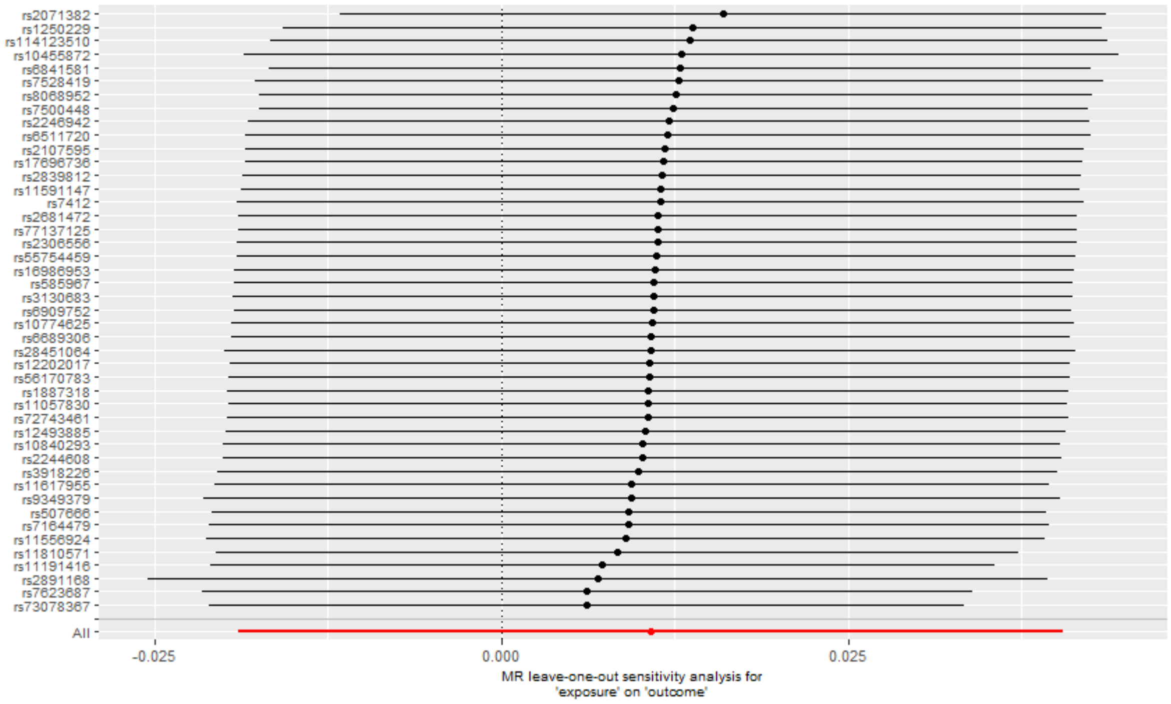

CAD Forest Plots (10^{-6})

EAS

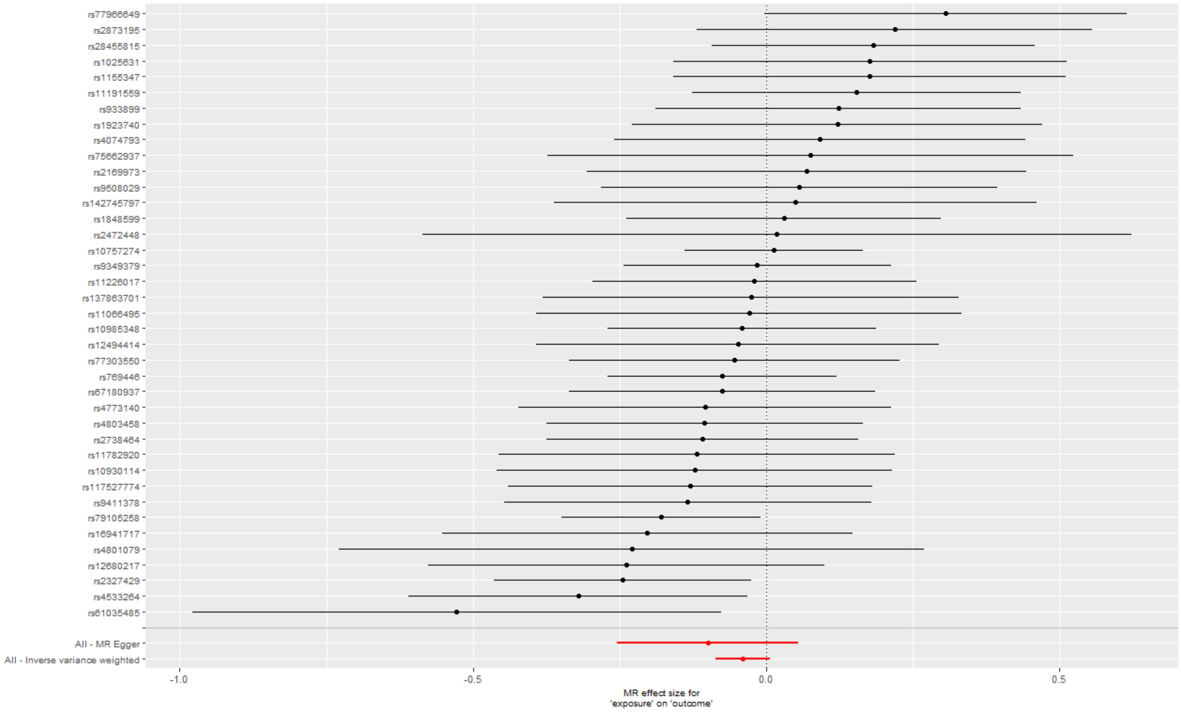

EUR

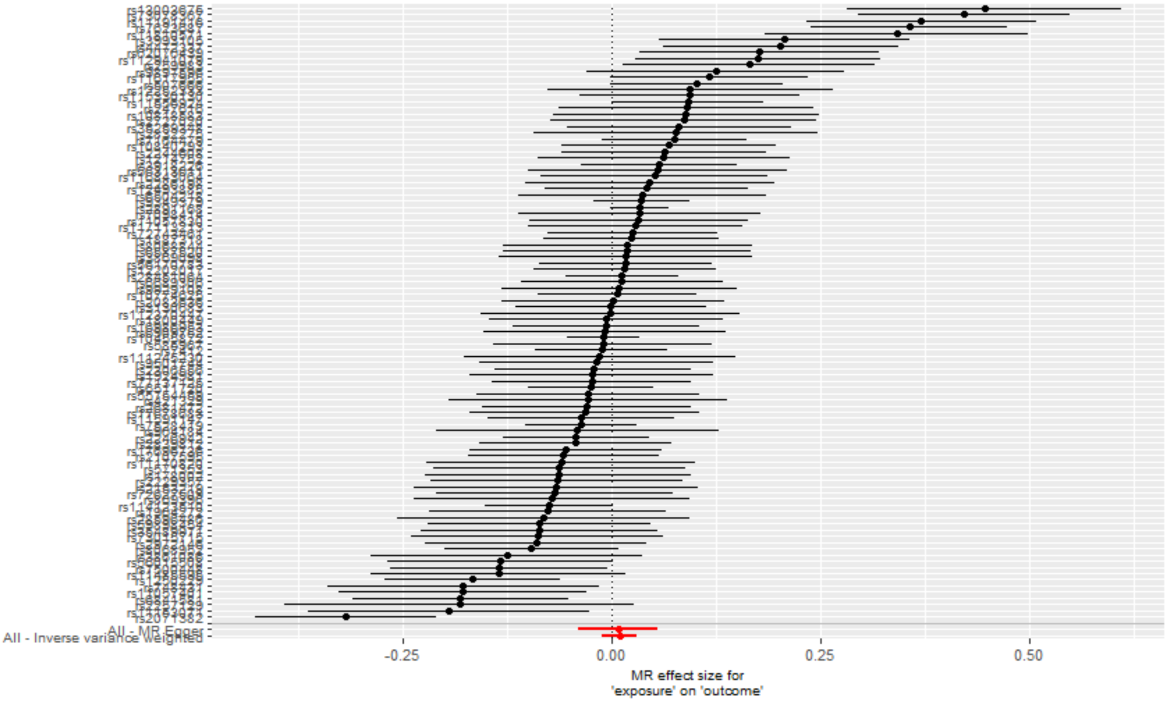

AFR

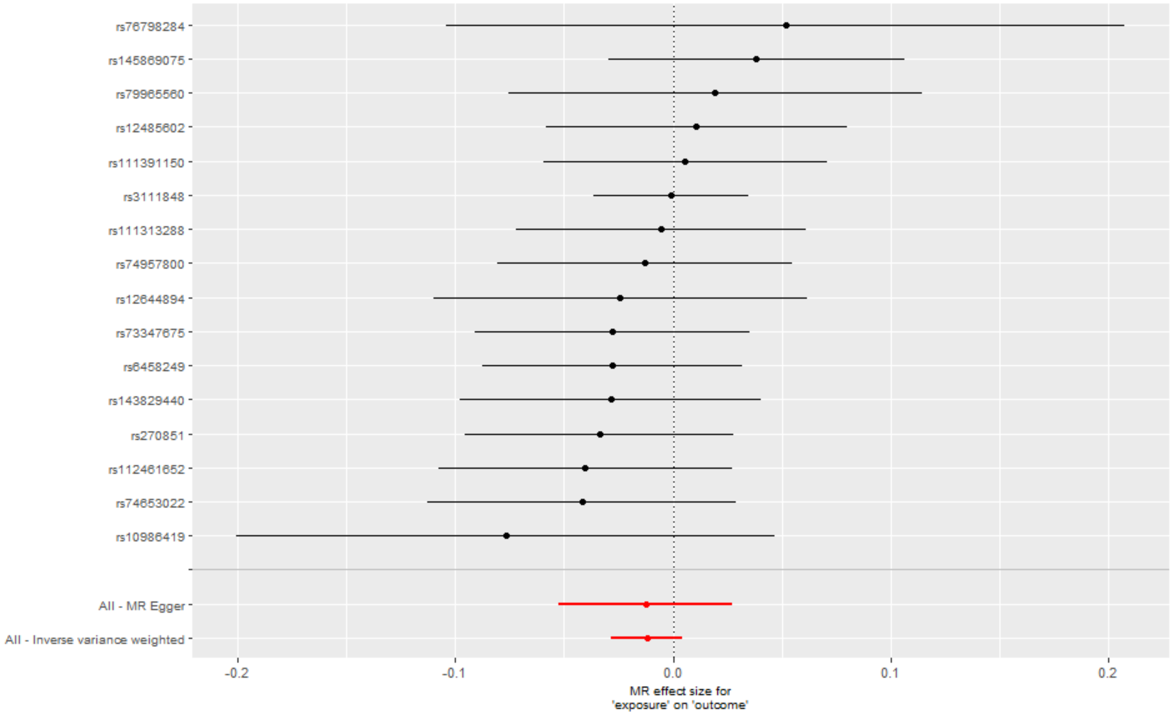

CAD Forest Plots (10^{-8})

EAS

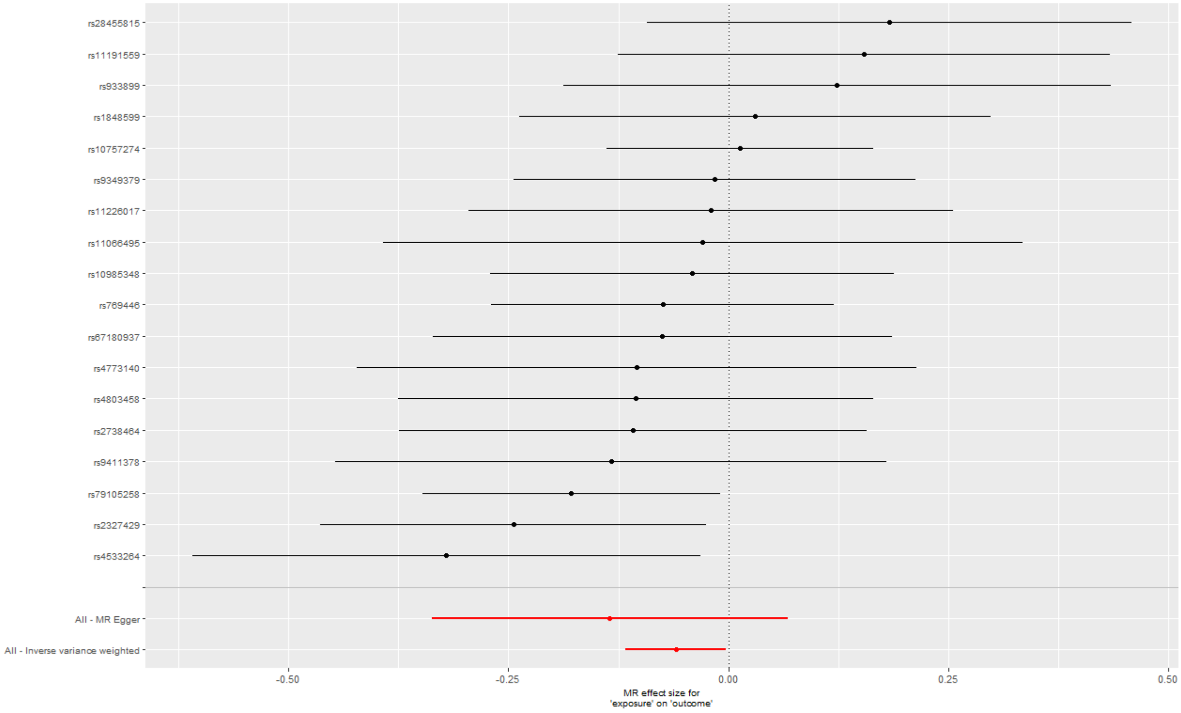

EUR

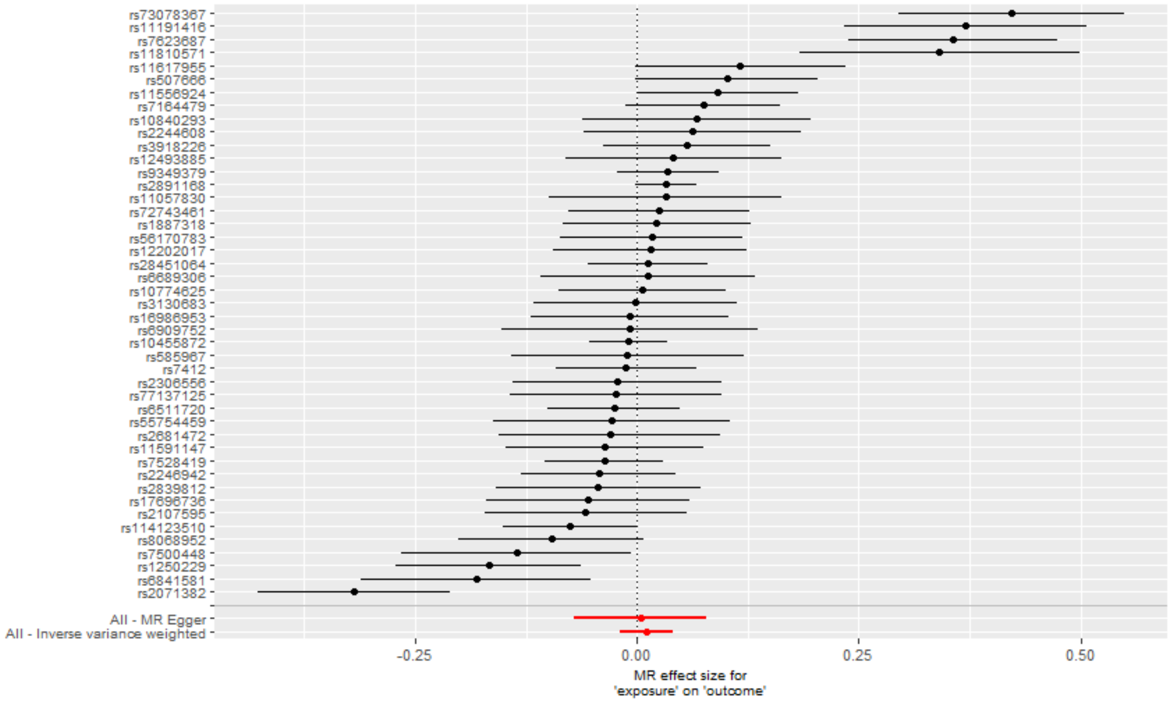

CAD Funnel Plots (10^{-6})

EAS

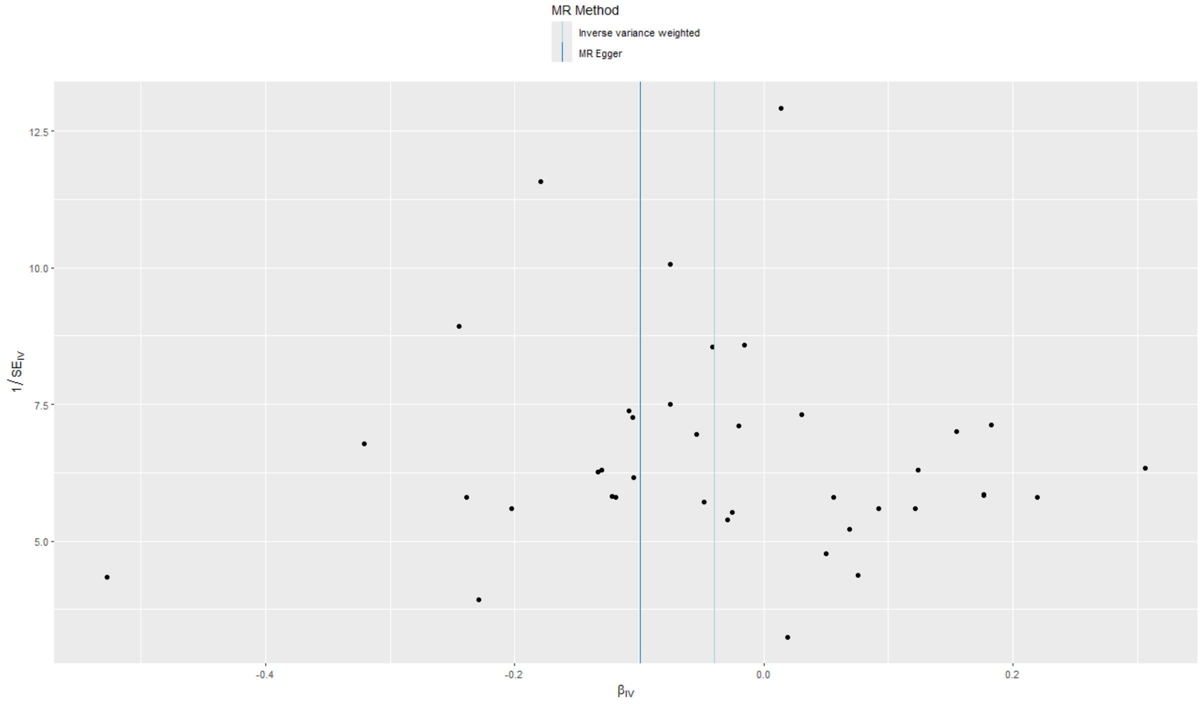

EUR

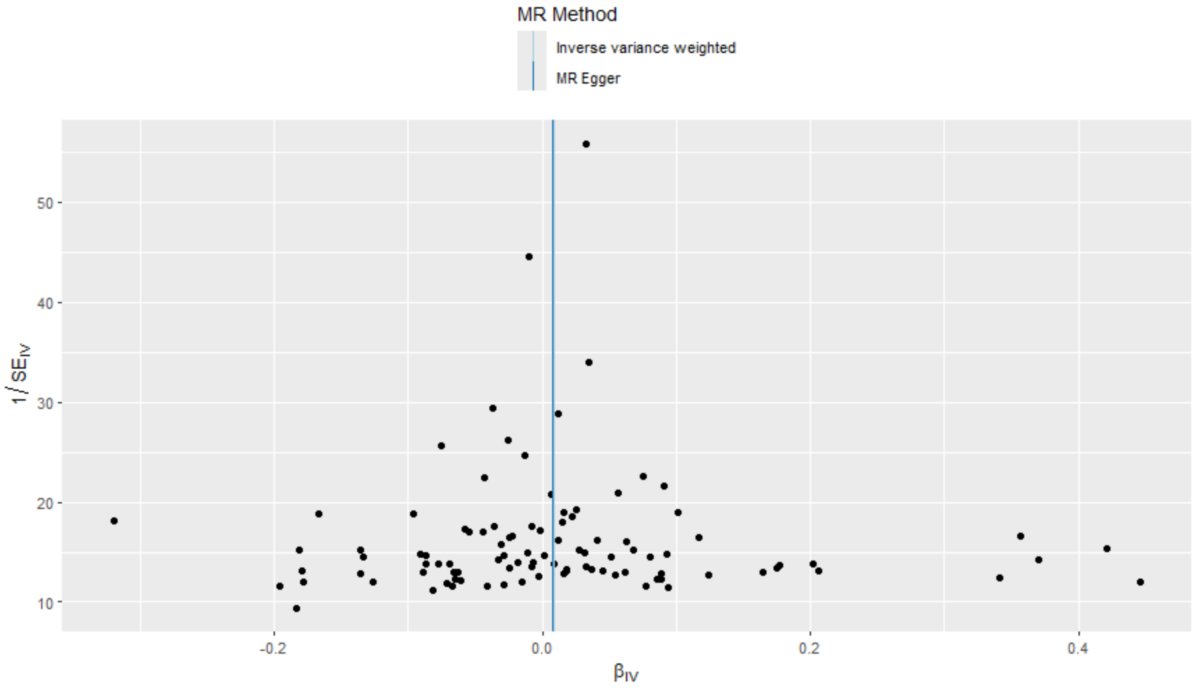
MR Method
Inverse variance weighted
MR Egger
50
40
30
20
10
-0.2
0.0
0.2
0.4
$1/SE_{IV}$
β_{IV}

AFR

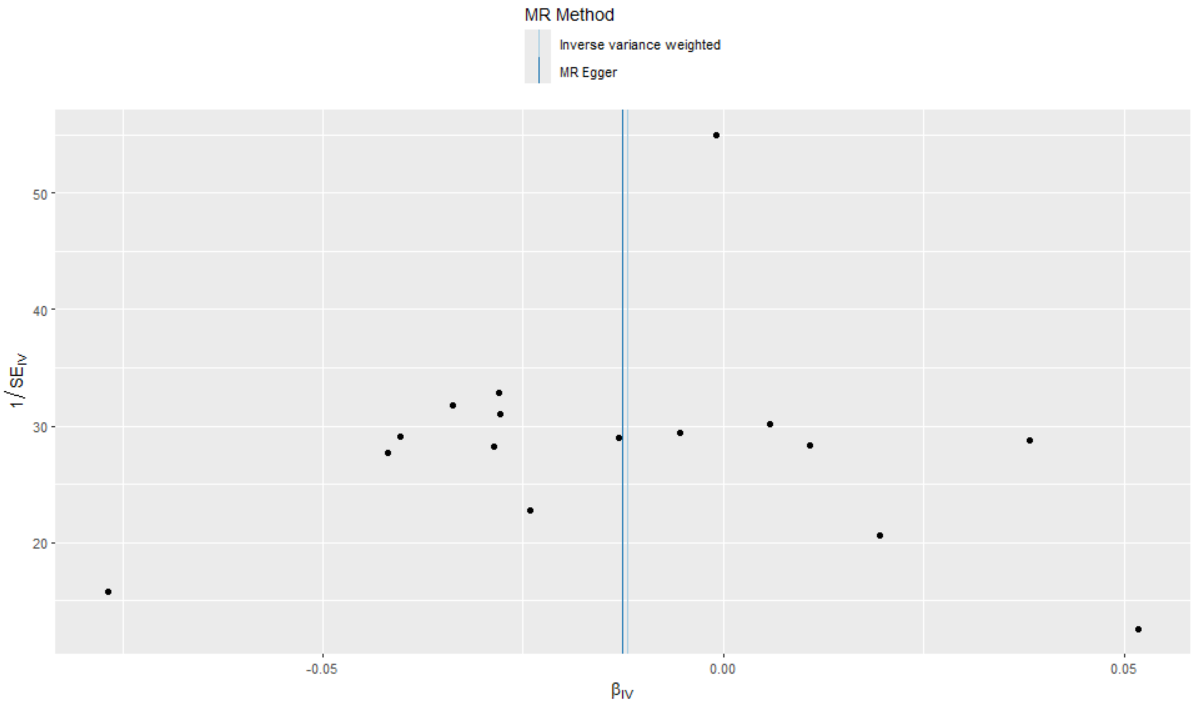
MR Method
Inverse variance weighted
MR Egger
50
40
30
20
-0.05
0.00
0.05
$1/SE_{IV}$
β_{IV}

CAD Funnel Plots (10^{-8})

EAS

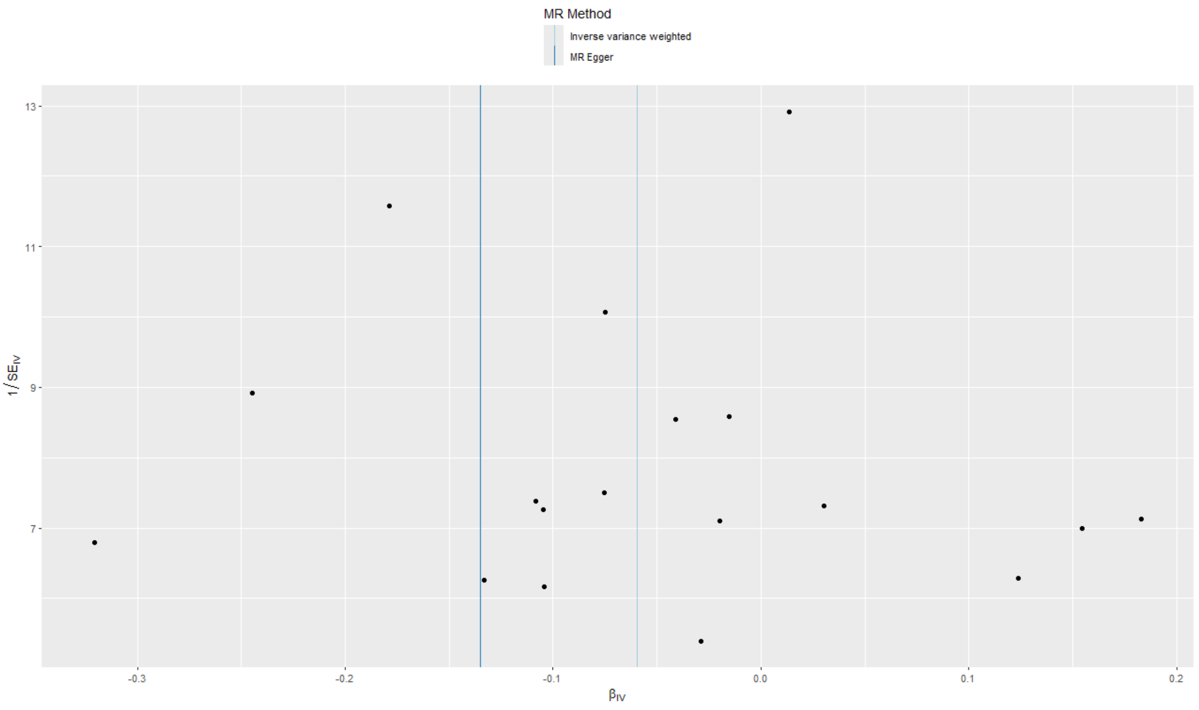

EUR

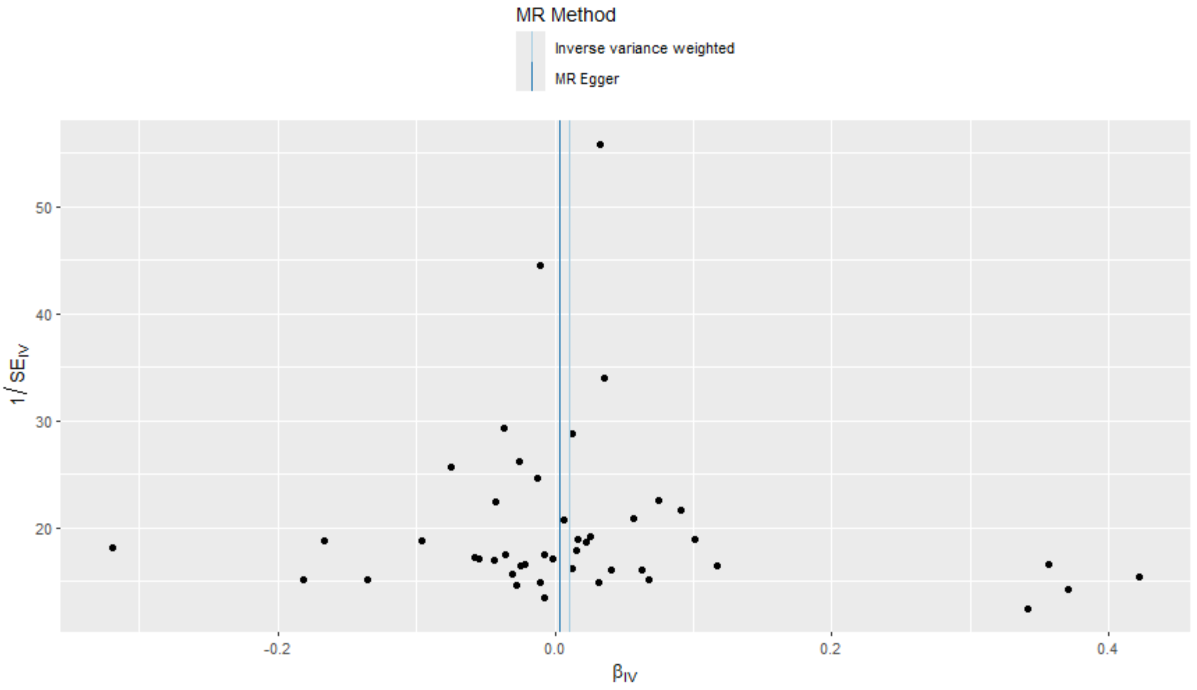

Supplement: Supplementary file 10 — Data S10: tmi70051‐sup‐0010‐supinfo.pdf. [file TMI-31-80-s003.pdf]
