# Supplementary material for: Causal Relationships Between Major Depressive Disorder and Coronary Artery Disease Across Diverse Populations: A Bidirectional Mendelian Randomisation Study
Source: Trop Med Int Health. 2025 Oct 15;31(1):80–90. doi: 10.1111/tmi.70051 (PMC12775895; doi:10.1111/tmi.70051)
Supplement: Supplementary file 11 — Data S11: tmi70051‐sup‐0011‐supinfo.pdf. [file TMI-31-80-s004.pdf]

STROBE-MR checklist of recommended items to address in reports of Mendelian randomization studies[1 2]

Item No.	Section	Checklist item	Page No.	Relevant text from manuscript
1	**TITLE and ABSTRACT**	Indicate Mendelian randomization (MR) as the study's design in the title and/or the abstract if that is a main purpose of the study	1-3	Title Page and Abstract section<br>*Lines 2-3*<br>Causal Relationships Between Major Depressive Disorder and Coronary Artery Disease Across Diverse Populations: A Bidirectional Mendelian Randomization Study<br><br>*Lines 25-54*<br><br>Background<br>Coronary artery disease (CAD) remains the leading cause of cardiovascular mortality worldwide, with a disproportionate burden in low- and middle-income countries. While observational studies have established a bidirectional relationship between depression and CAD, the underlying genetic basis of this association remains unclear, particularly in underrepresented diverse-ancestry populations. Establishing whether this relationship is causal and whether it differs by ancestry, is critical for informing targeted and equitable prevention strategies.<br>Methodology<br>This study employed a bidirectional two-sample Mendelian Randomization (MR) framework to investigate the causal relationship between major depressive disorder (MDD) and CAD across East Asian, European, and African populations. Using summary statistics from large-scale genome-wide association studies (GWAS), we assessed both the direction and strength of genetic associations between MDD and CAD. Sensitivity analyses were conducted to test the robustness of the findings.<br>Results<br>Genetically predicted MDD was associated with increased CAD risk in European populations, confirming a causal effect consistent with prior evidence. In East Asians, we found a potentially false-positive result suggesting an inverse association between genetic liability to CAD and MDD (IVW OR = 0.942; p = 0.040), which was not supported across MR sensitivity analyses and did not hold true after Bonferroni correction. In African ancestry populations, no significant causal effects were detected in either direction, due to wide confidence intervals resulting from limited sample sizes.<br>Interpretation<br>Our findings provide evidence for a causal relationship between depression and CAD in Europeans and suggest potential ancestry-specific variation in this relationship, with a novel inverse association between CAD and MDD observed in

				East Asians requiring further replication and validation. These results highlight how limited data availability in underrepresented populations constrains causal inference, underscoring the need for more inclusive genetic research to inform equitable, population-relevant public health strategies.
	**INTRODUCTION**			
2	**Background**	Explain the scientific background and rationale for the reported study. What is the exposure? Is a potential causal relationship between exposure and outcome plausible? Justify why MR is a helpful method to address the study question	4-5	Introduction section<br>*Lines 71-101*<br>"Globally, coronary artery disease (CAD) alone accounts for 42% of CVD-related deaths, placing immense strain on healthcare systems, particularly in resource-limited settings (1-4). Cardiovascular diseases (CVDs) remain the leading cause of mortality worldwide, with a disproportionate burden in low- and middle-income countries (LMICs) (5-6). Between 2008 and 2017, high-income countries produced 81.1% of global CVD research but accounted for just 8.1% of CVD-related disability-adjusted life-year (DALY) losses and 8.5% of deaths. In contrast, LMICs, where the burden of CVD is highest, generated only 2.8% of research despite accounting for 59.5% and 57.1% of global CVD DALY losses and death rates respectively (7).<br>Observational studies have reported a bidirectional association between major depressive disorder (MDD) and CAD, with depression linked to increased risk of cardiac events, and CAD associated with higher prevalence of depressive symptoms (4, 8-11). For example, a study by Lichtman and colleagues found that depression prevalence among CAD patients ranges from 15% to 30%, nearly two to three times higher than in the general population, while a separate study reported that patients with major depression faced nearly double the risk of cardiac mortality over a five-year period (12-13). Despite increasing recognition of this association, much of the evidence comes from European ancestry populations, with limited data from LMICs and other underrepresented groups (14-16). In many LMICs, depression remains underdiagnosed and undertreated due to stigma, insufficient mental health infrastructure, and limited access to care (17-18). As a result, the presence and directionality of the association between CAD and MDD in these populations remains unclear.<br>Mendelian Randomization (MR) provides a robust framework for reducing biases from confounding and reverse causation, providing insights into the biological mechanisms underlying disease risk (16, 19-22). While recent MR studies have investigated the bidirectional relationship between depression and cardiovascular diseases, findings consistently suggest that genetically predicted depression increases the risk of CAD, whereas the reverse direction — from CAD to depression — has shown null or inconclusive results (23–25). However, these analyses have been conducted predominantly in populations of European ancestry, limiting the generalisability of their findings. Given the known differences in genetic architecture and environmental exposures, expanding MR research to include diverse populations is critical to ensure that genetic insights translate into meaningful public health interventions globally (23, 26-27)."

3	**Objectives**	State specific objectives clearly, including pre-specified causal hypotheses (if any). State that MR is a method that, under specific assumptions, intends to estimate causal effects	5	Introduction section<br>*Lines 103-107*<br>"This study employs a bidirectional two-sample MR approach to investigate the causal relationship between major depressive disorder (MDD) and CAD across multiple ancestry groups. By identifying population-specific genetic determinants of MDD and CAD, this work aims to improve our understanding of the biological mechanisms underlying these conditions in diverse populations and contribute to more inclusive genetic research."
	**METHODS**			
4	**Study design and data sources**	Present key elements of the study design early in the article. Consider including a table listing sources of data for all phases of the study. For each data source contributing to the analysis, describe the following:	8	Figure 2<br>*Lines 159-163*
	a)	Setting: Describe the study design and the underlying population, if possible. Describe the setting, locations, and relevant dates, including periods of recruitment, exposure, follow-up, and data collection, when available.	Supplementary Material	Supplementary material section<br>*Supplementary material 1: data sources table*
	b)	Participants: Give the eligibility criteria, and the sources and methods of selection of participants. Report the sample size, and whether any power or sample size calculations were carried out prior to the main analysis	Supplementary Material	Supplementary material section<br>*Supplementary material 1: data sources table*
	c)	Describe measurement, quality control and selection of genetic variants	7	Methods section – Study Design<br>*Lines 139-157*<br>"The analytical framework for this study is depicted in Figure 2. Instrumental variables (IVs) were selected based on the three core MR assumptions outlined earlier. In the forward MR (MDD-to-CAD), IVs were selected from a GWAS of MDD, while in the reverse MR (CAD-to-MDD), IVs were selected from a GWAS of CAD. A genome-wide significance threshold of $P<5\times10^{-8}$ was applied to select IVs for the exposures; however, when fewer than ten variants remained after clumping, suggestive thresholds of $P<5\times10^{-}7$ and $P<5\times10^{-6}$ were instead applied.<br>Clumping was performed ($R^2 < 0.01$, 500kb window) to retain only independent IVs and avoid bias due to linkage disequilibrium (LD), where nearby genetic variants are inherited together due to their physical proximity on the genome. This step ensures that the selected IVs represent an independent single nucleotide polymorphism (SNP) rather than correlated variants, which reduces bias and overestimation of causal effects (32).<br>After selecting SNPs associated with the exposure, we identified these variants in the GWAS of the respective outcome traits — CAD for the forward MR and MDD

			for the reverse MR — to extract their corresponding allele frequencies and effect estimates. This ensured that the same genetic variants were assessed in both datasets, allowing for a valid two-sample MR analysis. In cases where an IV was absent from the outcome GWAS, a proxy SNP in high LD ($r^2 > 0.8$) was identified using ancestry-specific genotype data from Phase 3 Version 5 of the 1000 Genomes Project. Proxies were determined using the online tool SNiPA, ensuring that ancestry specificity was maintained across all analyses (33).”
d)	For each exposure, outcome, and other relevant variables, describe methods of assessment and diagnostic criteria for diseases	8-9	Methods section – Data sources<br>*Lines 166-180*<br>“Genetic instruments for MDD were sourced from ancestry-stratified GWAS conducted by the Psychiatric Genomics Consortium (PCG). Depression was defined based on a combination of self-reports, structured clinical interviews, symptom-based and medical health records. Ancestry-specific GWAS included individuals of African, East Asian, South Asian, and European descent. For this analysis, summary statistics included the PGC multi-ancestry GWAS of MDD, which incorporated individuals of African ancestry, a targeted GWAS of depression in East Asian populations, and a large-scale European GWAS that expanded upon previous multi-ancestry findings (24–25, 27).<br>Summary statistics for CAD were obtained from the GWAS Catalog and UK Biobank. CAD was defined based on medical records and clinical diagnoses within hospital-based registries and research cohorts. Because of this, the specific exposure phenotype derived by each GWAS was broadly defined, with CAD, ischemic heart disease, myocardial infarction and angina all included within the respective GWAS. Sources included the Coronary Artery Disease Genome-Wide Replication and Meta-analysis plus the Coronary Artery Disease consortium, Japan Biobank and UK Biobank (34-36). The summary information of each selected data source is provided in Supplementary Material 1.”
e)	Provide details of ethics committee approval and participant informed consent, if relevant	11	Methods section – Ethical Approval<br>*Lines 233-235*<br>“All GWAS datasets used in this study have obtained ethical approval and informed consent from participants. Since this study exclusively utilized publicly available summary-level data, no additional ethical approval was required.”

5	**Assumptions**	Explicitly state the three core IV assumptions for the main analysis (relevance, independence and exclusion restriction) as well assumptions for any additional or sensitivity analysis	5	Methods – Mendelian Randomization Methodology<br>*Lines 112-117*<br>"There are three core instrumental variable assumptions which need to be held true in an MR study to ensure valid causal inference: (1) the genetic variant must be strongly associated with the exposure of interest; (2) the genetic variant must not be associated with any confounders that influence both the exposure and outcome; and (3) the genetic variant must influence the outcome solely through the exposure, without any alternative pathways (28-30). Violations of these assumptions can bias effect estimates and limit their interpretability (31)."
6	**Statistical methods: main analysis**	Describe statistical methods and statistics used		
	a)	Describe how quantitative variables were handled in the analyses (i.e., scale, units, model)	9-11	Methods section – Statistical Analysis<br>*Lines 190-230*<br>"After identifying significant SNPs for each trait, we harmonized the effect alleles to ensure consistency in the direction of effect estimates across both exposure and outcome datasets. The primary bidirectional MR analysis was performed using the IVW approach under a fixed-effects model, which provides the most precise causal estimate when all IVs are valid. Forward (MDD-to-CAD) and reverse (CAD-to-MDD) MR analyses were conducted separately. As both MDD and CAD are treated as binary traits in the underlying genetic studies, effect estimates were reported as odds ratios (ORs), and no unit transformation was required. All analyses were conducted in R (version 4.3.2) using the TwoSampleMR (0.6.8) and MendelianRandomization (0.10.0) packages (37-40).<br>To assess the robustness of our findings and evaluate potential violations of MR assumptions, we performed a series of sensitivity analyses (Supplementary Material 4-10). The MR-Steiger directionality test was used to confirm whether the selected genetic instruments explained more variance in the exposure than in the outcome, supporting the assumption that the exposure precedes the outcome. A failure in the MR-Steiger test suggests possible misspecification of causality, where the instruments may be more strongly associated with the outcome than the exposure (38, 41).<br>To address potential violations of the exclusion restriction assumption, which states that genetic variants should only influence the outcome through the exposure, we applied MR-Egger regression and the weighted median estimator. These methods help mitigate the impact of pleiotropy, a key concern in MR studies where genetic variants may affect the outcome through alternative biological pathways. MR-Egger accounts for directional pleiotropy by allowing for a non-zero intercept, while the weighted median provides robust causal estimates even if up to 50% of the instruments are invalid (41–43). We also implemented the MR-PRESSO method to detect and correct for pleiotropic outliers, reducing the risk that observed effects were driven by invalid instruments (44).<br>We further assessed the strength of the genetic instruments using the F-statistic, which evaluates the strength of association between each SNP and the exposure. F-statistics greater than 10 were considered indicative of sufficiently strong

			instruments, reducing the likelihood of weak instrument bias that could attenuate results toward the null (45). To evaluate the consistency of causal estimates across individual SNPs, we assessed heterogeneity using Cochran's Q statistic and I^2 statistics. Diagnostic tools were also applied, including forest plots, funnel plots, and leave-one-out analyses, to visually inspect the influence of individual SNPs on the overall effect estimate.<br><br>Finally, we conducted statistical power calculations based on the available sample sizes for each trait. We estimated the minimum detectable ORs for each direction of the MR analyses at 80% power, across a range of plausible values representing the proportion of variance in the exposure explained by the genetic instruments (R^2); an 80% power threshold was considered sufficient to detect potentially meaningful effects (40). This approach accounts for the uncertainty inherent in power estimation for MR studies, where assumptions about true effect size and instrument strength can vary, and offers a more transparent alternative to static a priori or post hoc power estimates.<br><br>To account for multiple comparisons in the bidirectional analysis, we applied a Bonferroni correction to control for the risk of type I error."
b)	Describe how genetic variants were handled in the analyses and, if applicable, how their weights were selected	7	Methods section – Study Design<br><br>Lines 139-157<br><br>"The analytical framework for this study is depicted in Figure 2. Instrumental variables (IVs) were selected based on the three core MR assumptions outlined earlier. In the forward MR (MDD-to-CAD), IVs were selected from a GWAS of MDD, while in the reverse MR (CAD-to-MDD), IVs were selected from a GWAS of CAD. A genome-wide significance threshold of $P<5\times10^{-8}$ was applied to select IVs for the exposures; however, when fewer than ten variants remained after clumping, suggestive thresholds of $P<5\times10^{-}$7 and $P<5\times10^{-6}$ were instead applied.<br><br>Clumping was performed ($R^2 < 0.01$, 500kb window) to retain only independent IVs and avoid bias due to linkage disequilibrium (LD), where nearby genetic variants are inherited together due to their physical proximity on the genome. This step ensures that the selected IVs represent an independent single nucleotide polymorphism (SNP) rather than correlated variants, which reduces bias and overestimation of causal effects (32).<br><br>After selecting SNPs associated with the exposure, we identified these variants in the GWAS of the respective outcome traits — CAD for the forward MR and MDD for the reverse MR — to extract their corresponding allele frequencies and effect estimates. This ensured that the same genetic variants were assessed in both datasets, allowing for a valid two-sample MR analysis. In cases where an IV was absent from the outcome GWAS, a proxy SNP in high LD ($r^2 > 0.8$) was identified using ancestry-specific genotype data from Phase 3 Version 5 of the 1000 Genomes Project. Proxies were determined using the online tool SNiPA, ensuring that ancestry specificity was maintained across all analyses (33)."

		c) Describe the MR estimator (e.g. two-stage least squares, Wald ratio) and related statistics. Detail the included covariates and, in case of two-sample MR, whether the same covariate set was used for adjustment in the two samples	9-10	Methods section – Statistical Analysis<br>*Lines 191-197*<br>"The primary bidirectional MR analysis was performed using the IVW approach under a fixed-effects model, which provides the most precise causal estimate when all IVs are valid. Forward (MDD-to-CAD) and reverse (CAD-to-MDD) MR analyses were conducted separately. As both MDD and CAD are treated as binary traits in the underlying genetic studies, effect estimates were reported as odds ratios (ORs), and no unit transformation was required. All analyses were conducted in R (version 4.3.2) using the TwoSampleMR (0.6.8) and MendelianRandomization (0.10.0) packages (37-40)."
		d) Explain how missing data were addressed	9-10	Methods section – Statistical Analysis<br>*Lines 190-196*<br>"After identifying significant SNPs for each trait, we harmonized the effect alleles to ensure consistency in the direction of effect estimates across both exposure and outcome datasets. The primary bidirectional MR analysis was performed using the IVW approach under a fixed-effects model, which provides the most precise causal estimate when all IVs are valid. Forward (MDD-to-CAD) and reverse (CAD-to-MDD) MR analyses were conducted separately. As both MDD and CAD are treated as binary traits in the underlying genetic studies, effect estimates were reported as odds ratios (ORs), and no unit transformation was required."
		e) If applicable, indicate how multiple testing was addressed	11	Methods section – Statistical Analysis<br>*Lines 229-230*<br>"To account for multiple comparisons in the bidirectional analysis, we applied a Bonferroni correction to control for the risk of type I error.
7	**Assessment of assumptions**	Describe any methods or prior knowledge used to assess the assumptions or justify their validity	10	Methods section – Statistical Analysis<br>*Lines 198-213*<br>"To assess the robustness of our findings and evaluate potential violations of MR assumptions, we performed a series of sensitivity analyses (Supplementary Material 4-10). The MR-Steiger directionality test was used to confirm whether the selected genetic instruments explained more variance in the exposure than in the outcome, supporting the assumption that the exposure precedes the outcome. A failure in the MR-Steiger test suggests possible misspecification of causality, where the instruments may be more strongly associated with the outcome than the exposure (38, 41).<br>To address potential violations of the exclusion restriction assumption, which states that genetic variants should only influence the outcome through the exposure, we applied MR-Egger regression and the weighted median estimator. These methods help mitigate the impact of pleiotropy, a key concern in MR studies where genetic variants may affect the outcome through alternative biological pathways. MR-Egger

				accounts for directional pleiotropy by allowing for a non-zero intercept, while the weighted median provides robust causal estimates even if up to 50% of the instruments are invalid (41–43). We also implemented the MR-PRESSO method to detect and correct for pleiotropic outliers, reducing the risk that observed effects were driven by invalid instruments (44).”
8	**Sensitivity analyses and additional analyses**	Describe any sensitivity analyses or additional analyses performed (e.g. comparison of effect estimates from different approaches, independent replication, bias analytic techniques, validation of instruments, simulations)	10-11	Methods section – Statistical Analysis<br><br>*Lines 198-228*<br><br>“To assess the robustness of our findings and evaluate potential violations of MR assumptions, we performed a series of sensitivity analyses (Supplementary Material 4-10). The MR-Steiger directionality test was used to confirm whether the selected genetic instruments explained more variance in the exposure than in the outcome, supporting the assumption that the exposure precedes the outcome. A failure in the MR-Steiger test suggests possible misspecification of causality, where the instruments may be more strongly associated with the outcome than the exposure (38, 41).<br><br>To address potential violations of the exclusion restriction assumption, which states that genetic variants should only influence the outcome through the exposure, we applied MR-Egger regression and the weighted median estimator. These methods help mitigate the impact of pleiotropy, a key concern in MR studies where genetic variants may affect the outcome through alternative biological pathways. MR-Egger accounts for directional pleiotropy by allowing for a non-zero intercept, while the weighted median provides robust causal estimates even if up to 50% of the instruments are invalid (41–43). We also implemented the MR-PRESSO method to detect and correct for pleiotropic outliers, reducing the risk that observed effects were driven by invalid instruments (44).<br><br>We further assessed the strength of the genetic instruments using the F-statistic, which evaluates the strength of association between each SNP and the exposure. F-statistics greater than 10 were considered indicative of sufficiently strong instruments, reducing the likelihood of weak instrument bias that could attenuate results toward the null (45). To evaluate the consistency of causal estimates across individual SNPs, we assessed heterogeneity using Cochran’s Q statistic and I^2 statistics. Diagnostic tools were also applied, including forest plots, funnel plots, and leave-one-out analyses, to visually inspect the influence of individual SNPs on the overall effect estimate.<br><br>Finally, we conducted statistical power calculations based on the available sample sizes for each trait. We estimated the minimum detectable ORs for each direction of the MR analyses at 80% power, across a range of plausible values representing the proportion of variance in the exposure explained by the genetic instruments (R^2); an 80% power threshold was considered sufficient to detect potentially meaningful effects (40). This approach accounts for the uncertainty inherent in power estimation for MR studies, where assumptions about true effect size and instrument strength can vary, and offers a more transparent alternative to static a priori or post hoc power estimates.”

9	**Software and pre-registration**				
		a)	Name statistical software and package(s), including version and settings used	10	Methods section – Statistical Analysis<br>*Lines 196-197*<br>“All analyses were conducted in R (version 4.3.2) using the TwoSampleMR (0.6.8) and MendelianRandomization (0.10.0) packages (37-40).”
		b)	State whether the study protocol and details were pre-registered (as well as when and where)	23-24	Data Sharing Statement section<br>Lines 476-480<br>“The genome-wide association summary statistics data used in this study are publicly available at https://www.ebi.ac.uk/gwas/downloads/summary-statistics and https://pgc.unc.edu/for-researchers/download-results/. All software programs used in this paper are listed and referenced in the Methods. The full coding script used to perform the analysis is available from the corresponding authors upon request.”
	**RESULTS**				
10	**Descriptive data**				
		a)	Report the numbers of individuals at each stage of included studies and reasons for exclusion. Consider use of a flow diagram	N/A	
		b)	Report summary statistics for phenotypic exposure(s), outcome(s), and other relevant variables (e.g. means, SDs, proportions)	Supplementary Material	Supplementary material section<br>*Supplementary material 1: data sources table*<br>*Supplementary material 2: number of SNPs*
		c)	If the data sources include meta-analyses of previous studies, provide the assessments of heterogeneity across these studies	N/A	

		d) For two-sample MR:<br>i. Provide justification of the similarity of the genetic variant-exposure associations between the exposure and outcome samples<br>ii. Provide information on the number of individuals who overlap between the exposure and outcome studies	7; 9	Methods section – Study Design<br>*Lines 139-157*<br>"The analytical framework for this study is depicted in Figure 2. Instrumental variables (IVs) were selected based on the three core MR assumptions outlined earlier. In the forward MR (MDD-to-CAD), IVs were selected from a GWAS of MDD, while in the reverse MR (CAD-to-MDD), IVs were selected from a GWAS of CAD. A genome-wide significance threshold of $P<5\times10^{-8}$ was applied to select IVs for the exposures; however, when fewer than ten variants remained after clumping, suggestive thresholds of $P<5\times10^{-}7$ and $P<5\times10^{-6}$ were instead applied.<br>Clumping was performed ($R^2 < 0.01$, 500kb window) to retain only independent IVs and avoid bias due to linkage disequilibrium (LD), where nearby genetic variants are inherited together due to their physical proximity on the genome. This step ensures that the selected IVs represent an independent single nucleotide polymorphism (SNP) rather than correlated variants, which reduces bias and overestimation of causal effects (32).<br>After selecting SNPs associated with the exposure, we identified these variants in the GWAS of the respective outcome traits — CAD for the forward MR and MDD for the reverse MR — to extract their corresponding allele frequencies and effect estimates. This ensured that the same genetic variants were assessed in both datasets, allowing for a valid two-sample MR analysis. In cases where an IV was absent from the outcome GWAS, a proxy SNP in high LD ($r^2 > 0.8$) was identified using ancestry-specific genotype data from Phase 3 Version 5 of the 1000 Genomes Project. Proxies were determined using the online tool SNiPA, ensuring that ancestry specificity was maintained across all analyses (33)."<br><br>Methods section – Data Sources<br>*Lines 182-187*<br>"To ensure the independence of exposure and outcome data sources within ancestry groups, as required by the two-sample MR framework, we undertook a review of the study cohorts contributing to both the MDD and CAD GWAS. Published methodologies, consortium documentation, and supplementary materials were examined to identify any potential sample overlap. Based on this review, no sample overlap was identified for African, East Asian, or European populations; for the latter, outcome data excluding UK Biobank participants was used to avoid duplication."
11	**Main results**			
		a) Report the associations between genetic variant and exposure, and between genetic variant and outcome, preferably on an interpretable scale	Supplementary Material	Supplementary material section<br>*Supplementary material 3: MR results*<br>*Supplementary material 6: SNP information*

		b)	Report MR estimates of the relationship between exposure and outcome, and the measures of uncertainty from the MR analysis, on an interpretable scale, such as odds ratio or relative risk per SD difference	11-12	Results section – Bidirectional Two-Sample Mendelian Randomization Analysis<br>*Lines 240-253*<br>“MR analysis in Europeans showed evidence of a causal effect of MDD on CAD, where a genetically predicted increase in liability to MDD was associated with 31% higher odds of CAD (IVW OR = 1.314; 95% CI, 1.218–1.419; P < 0.001). In the reverse direction, no association was observed between genetically predicted liability to CAD and risk of MDD (IVW OR = 1.011; 95% CI, 0.981–1.042; P = 0.477), and findings remained consistent when more relaxed p-value thresholds were applied (Figure 3; Supplementary Material 3).<br>In East Asians, the IVW estimate suggested a potential inverse association between CAD on MDD (IVW OR = 0.942; 95% CI, 0.890–0.997; P = 0.040). However, this association was not supported by alternative MR methods or sensitivity analyses and may represent a false-positive finding, as it did not remain statistically significant after Bonferroni correction for multiple testing across the bidirectional analyses (adjusted significance threshold: P < 0.0125).Moreover, the association was not observed when more relaxed p-value thresholds were applied for instrument selection (IVW OR = 0.961; 95% CI, 0.919–1.006; P = 0.091). No evidence of a causal effect was detected in the reverse direction in East Asians, nor in either direction in African populations (Figure 3).”
		c)	If relevant, consider translating estimates of relative risk into absolute risk for a meaningful time period	N/A	
		d)	Consider plots to visualize results (e.g. forest plot, scatterplot of associations between genetic variants and outcome versus between genetic variants and exposure)	12-14	Figure 3<br>*Lines 255-264*
12	**Assessment of assumptions**				
		a)	Report the assessment of the validity of the assumptions	Supplementary Material	Supplementary material section<br>*Supplementary material 6: SNP information*<br>*Supplementary material 7*: Minimum detectable OR at 80% power<br>*Supplementary material 8*: Power Plots
		b)	Report any additional statistics (e.g., assessments of heterogeneity across genetic variants, such as I^2, Q statistic or E-value)	Supplementary Material	Supplementary material section<br>*Supplementary material 4: Heterogeneity analysis (Q-Statistic)*<br>*Supplementary material 5: MR-PRESSO analysis*

				Supplementary material 10: Leave-one-out analysis, Funnel plots and Forest plots<br><br>*Supplementary material 9: MR-Steiger results*
13	**Sensitivity analyses and additional analyses**			
		a) Report any sensitivity analyses to assess the robustness of the main results to violations of the assumptions	15-18	Results section – Statistical Power and Heterogeneity<br><br>*Lines 267-303*<br><br>"Power curves showed that, for a given R^2, the detectable OR at 80% power varied across populations due to differences in sample size. For instance, at $R^2 = 0.05$, the minimum detectable ORs in the forward MR were approximately 1.12 in Europeans, 1.13 in East Asians, and 1.70 in Africans. In the reverse direction, the corresponding ORs were approximately 1.02 in Europeans, 1.11 in East Asians, and 1.08 in Africans. At a higher instrument strength (e.g., $R^2 = 0.1$), the minimum detectable ORs in the forward MR decreased to 1.08 in Europeans, 1.09 in East Asians, and 1.50 in Africans, while in the reverse MR, the values were 1.08 in Europeans, 1.02 in East Asians, and 1.06 in Africans. These findings illustrate the lower sensitivity to modest effect sizes in smaller samples, particularly for African ancestry populations.<br><br>To evaluate the strength of the instrumental variables, we calculated the median F-statistics for each ancestry group using the R^2 values derived from the individual SNPs in our analysis (Supplementary Material 6). In the forward MR, with MDD as the exposure, European instruments were consistently strong ($F \geq 10$), with a median F-statistic of 15 at $P < 5\times10^{-8}$. In contrast, instruments for East Asian and African populations were weak ($F < 10$) across all p-value thresholds.<br><br>In the reverse MR, with CAD as the exposure, both European and East Asian instruments were strong, with a median F-statistic of 15 for East Asians at $P<5\times10^{-8}$. As with the forward MR, the median F-statistics for African populations remained weak, regardless of the p-value threshold.<br><br>Cochran's Q tests were used to evaluate heterogeneity among the IVs for each ancestry group at the genome-wide significance threshold of $P < 5\times10^{-8}$. In the forward MR, with MDD as the exposure, significant heterogeneity was observed among European instruments using both IVW (Q = 352.92, df = 197, P < 0.001) and MR-Egger methods (Q = 345.63, df = 196, P < 0.001). Heterogeneity could not be assessed in East Asians due to an insufficient number of variants, and no estimates were available for African ancestry populations due to the lack of instruments. In the reverse MR analysis, with CAD as the exposure, significant heterogeneity was again observed in Europeans (IVW Q = 213.44, df = 44, P = 0.000; MR-Egger Q = 213.23, df = 43, P = 0.000), while no evidence of heterogeneity was found in East Asians (IVW Q = 16.49, df = 17, P = 0.490; MR-Egger Q = 15.91, df = 16, P = 0.459). Heterogeneity statistics for African ancestry populations could not be calculated due to the lack of available instruments. Full heterogeneity results at both $P < 5\times10^{-8}$ and $P < 5\times10^{-6}$ thresholds are provided in Supplementary Material 4."

Results section – Sensitivity Analyses

Lines 306-340

”To assess the robustness of our MR results and check for any violations of key assumptions, we performed several sensitivity analyses (Table 1; Supplementary Material 4-10). The MR-Steiger test indicated a high failure rate in the forward direction (MDD as the exposure) for European populations (54% at $P < 5\times10^{-8}$; 60% at $P < 5\times10^{-6}$), suggesting that many instruments were more strongly associated with CAD than MDD. Failure rates were lower in Africans (47%) and East Asians (0%), indicating some population differences in instrument validity. In the reverse MR direction (CAD as the exposure), all instruments passed the MR-Steiger test across each of the populations.

[Table 1]

We compared IVW estimates with MR-Egger and weighted median models to evaluate consistency across methods. In Europeans, MR-Egger regression suggested directional pleiotropy at both thresholds (intercepts: 0.992 and 0.996, both $P < 0.001$), and MR-PRESSO detected significant global pleiotropy ($P < 2\times10^{-4}$) in all forward and reverse MR analyses. No pleiotropy was detected in East Asian or African populations.

MR-PRESSO identified significant global pleiotropy in both the forward and reverse analyses among Europeans ($P < 0.0002$), with several outlier SNPs detected in each direction. In the forward analysis, correction for three outliers yielded a substantially stronger effect estimate (OR = 0.29, $P = 9.28\times10^{-14}$), although the distortion test was not significant ($P = 0.738$), suggesting the correction did not materially alter the interpretation. In the reverse analysis, five outliers were removed, but the corrected estimate was negligible (OR = 2.83×10^{-4}, $P = 0.974$), although the distortion was significant ($P = 0.007$), indicating a marked influence of outliers on the original estimate. No outliers were identified in East Asians, and tests could not be conducted in Africans due to insufficient instruments.”

Table 1

Lines 320-325

		b) Report results from other sensitivity analyses or additional analyses	Supplementary Material	Supplementary material section<br>*Supplementary material 4: Heterogeneity analysis (Q-Statistic)*<br>*Supplementary material 5: MR-PRESSO analysis*<br>*Supplementary material 6: SNP information*<br>*Supplementary material 7*: Minimum detectable OR at 80% power<br>*Supplementary material 8*: Power Plots<br>*Supplementary material 10: Leave-one-out analysis, Funnel plots and Forest plots*<br>*Supplementary material 9: MR-Steiger results*
		c) Report any assessment of direction of causal relationship (e.g., bidirectional MR)	12-14	Figure 3<br>*Lines 255-264*
		d) When relevant, report and compare with estimates from non-MR analyses	N/A	
		e) Consider additional plots to visualize results (e.g., leave-one-out analyses)	Supplementary Material	Supplementary material section<br>*Supplementary material 10: Leave-one-out analysis, Funnel plots and Forest plots*
	**DISCUSSION**			
14	**Key results**	Summarize key results with reference to study objectives	18	Discussion section<br>*Lines 344-353*<br>"This study presents a large-scale bidirectional two-sample Mendelian randomization analysis to evaluate the causal relationship between MDD and CAD across East Asian, European, and African populations. Our results indicate a causal effect of genetically predicted MDD on increased CAD risk in Europeans, aligning with previous MR findings. In the reverse direction, we identified a weak inverse association between CAD and MDD in East Asians, with borderline significance (p = 0.040) in the primary IVW analysis. This suggests that while depression may increase the risk of heart disease in European populations, there is also preliminary evidence indicating that genetic liability to CAD might be associated with a slightly reduced risk of depression in East Asians. However, as

				this finding did not meet the Bonferroni-adjusted threshold, the results from this analysis require replication and further validation.”
15	**Limitations**	Discuss limitations of the study, taking into account the validity of the IV assumptions, other sources of potential bias, and imprecision. Discuss both direction and magnitude of any potential bias and any efforts to address them	21-22	Discussion section – Methodological Considerations and Limitations<br>*Lines 411-443*<br>“Several methodological considerations warrant attention. First, genetic instrument selection for East Asian and African populations relied on less stringent p-value thresholds and clumping parameters due to the limited availability of genome-wide significant SNPs, especially in the forward MR direction (MDD as the exposure). While this approach was necessary to increase power and enable bidirectional analyses, it may have introduced weak instrument bias, reduced the precision of causal estimates, and increased sensitivity to specific analytical choices, potentially attenuating true effects. Relaxing instrument selection criteria can also increase the risk of including variants with weaker associations to the exposure, which may bias MR estimates toward the null. This is especially relevant given that the resulting F-statistics for several analyses fell within borderline ranges, raising additional concerns about the strength of the instruments and the robustness of the causal inference.<br>Second, although we performed multiple sensitivity analyses, residual pleiotropy remains a concern. In the European dataset, MR-Egger intercept tests and MR-PRESSO analyses indicated horizontal pleiotropy, suggesting that some genetic variants may influence outcomes through pathways unrelated to the exposure. In East Asians, while the inverse association between CAD and MDD reached borderline significance in the primary IVW analysis, neither MR-Egger nor Weighted Median methods produced significant estimates, and confidence intervals were wide. This inconsistency suggests that the result may be vulnerable to pleiotropic bias and highlights the need for cautious interpretation.<br>Selection bias is another important limitation to consider. The GWAS datasets used in this study were derived from large biobank cohorts, many of which include volunteers who tend to be healthier and are less likely to have severe mental or physical health conditions compared to the general population (65-66). This may have biased effect estimates if the genetic determinants of depression and CAD differ between biobank participants and the general population. Recruitment strategies, healthcare access, and socioeconomic disparities are also likely to influence the demographics of individuals included in the respective datasets.<br>Finally, statistical power varied across populations. While the European and East Asian GWAS provided well-powered data for MR, the African CAD datasets were underpowered, limiting instrument strength and the interpretability of findings in that population. This highlights the need for larger, ancestrally diverse GWAS to enable more robust and generalisable multi-ancestry MR analyses.”

16	**Interpretation**			
	a)	Meaning: Give a cautious overall interpretation of results in the context of their limitations and in comparison with other studies	20	Discussion section<br>*Lines 392-408*<br>"Although novel, the inverse association observed is inconsistent with the null results reported in earlier MR studies, underscoring the need for replication using larger, well-powered datasets and pleiotropy-robust methods. Moreover, as this association did not meet the Bonferroni-corrected significance threshold, these findings should be considered preliminary and warrant further study to clarify whether this signal reflects true biological effects or methodological artifacts (14–15, 54–59).<br>More broadly, while MR is designed to mitigate confounding and reverse causation, the complex, multifactorial nature of both MDD and CAD necessitates interpretation of causal findings alongside broader epidemiological and clinical evidence. Shared biological pathways, such as systemic inflammation or physical inactivity, may contribute to both conditions, and psychiatric symptoms may only emerge clinically following a CAD event (60–62). Conversely, undiagnosed depression may precede CAD onset but only come to medical attention during cardiovascular assessment (63–64). Although such diagnostic patterns are unlikely to bias MR estimates, they remain important for understanding how these conditions present and progress. While MR offers valuable insight into underlying causal pathways, incorporating both genetic and non-genetic evidence is essential to fully capture ancestry-specific variation and inform equitable, population-relevant public health strategies."
	b)	Mechanism: Discuss underlying biological mechanisms that could drive a potential causal relationship between the investigated exposure and the outcome, and whether the gene-environment equivalence assumption is reasonable. Use causal language carefully, clarifying that IV estimates may provide causal effects only under certain assumptions	18-20	Discussion section<br>*Lines 356-391*<br>"Biological pathways such as systemic inflammation, hypothalamic–pituitary–adrenal axis dysregulation, and metabolic disruption are proposed mediators in this relationship (47–50). Behavioural factors, including physical inactivity, poor dietary habits, and smoking, which are more prevalent among individuals with depression, likely also contribute to increased CAD susceptibility. Taken together, these mechanisms highlight how depression may influence CAD risk through both physiological and behavioural pathways, underscoring the importance of integrating mental health into cardiovascular risk reduction frameworks.<br>In addition to confirming established risk pathways, this study also identified a novel potential association between genetic liability to CAD and a reduced risk of MDD in East Asian populations. The IVW analysis suggested a modest inverse association between CAD and MDD risk; however, this effect was close to the threshold for statistical significance and not supported by sensitivity analyses. Specifically, alternative MR methods such as the weighted median and MR-Egger produced non-significant estimates with wide confidence intervals. Although no outliers were identified by MR-PRESSO and instrument strength was adequate, albeit in the borderline range (F = 15), these findings raise uncertainty about the robustness of the result.<br>One possible explanation for the inverse association is subtle horizontal pleiotropy, where genetic variants independently influence both CAD and MDD through distinct pathways, potentially in opposing directions, thereby violating the MR

				assumption that genetic instruments affect the outcome only through the exposure. While MR-Egger did not detect significant directional pleiotropy, the borderline IVW estimate may still reflect unbalanced pleiotropic effects — such as genetic variants influencing intermediate traits like metabolic risk factors — which are not fully captured by standard sensitivity analyses.<br><br>Additionally, it remains possible that the instruments used do not fully capture the biological pathways linking CAD and MDD in East Asian ancestry individuals. Phenotypic heterogeneity, differences in sample characteristics, and environmental variation across cohorts, including population-specific gene–environment interactions, may contribute to the observed effect (51). In East Asian populations, differences in environmental exposures or lifestyle factors may modify the relationship between genetic liability to CAD and depression risk. In addition, variation in how depression is phenotypically defined across studies may complicate interpretation. Psychiatric symptoms are often underreported in some cultural contexts, potentially distorting genetic correlations, and clinical criteria for MDD diagnosis may differ across settings (52-53). As a result, the depression phenotype captured in East Asian GWAS may not be directly comparable to that in European cohorts, introducing additional heterogeneity into cross-population MR analyses.”
		c) Clinical relevance: Discuss whether the results have clinical or public policy relevance, and to what extent they inform effect sizes of possible interventions	22-23	Discussion section<br><br>*Lines 454-461*<br><br>“Future studies should prioritize expanding GWAS in non-European populations, as larger, ancestrally diverse samples will strengthen instruments, reduce bias, and enable more robust and interpretable MR analyses. Incorporating polygenic risk scores, gene–environment interaction models, and multi-omics approaches — such as transcriptomics, methylation, or metabolomics — will further clarify the biological pathways linking depression and CAD. Additionally, replicating and reassessing the inverse association between CAD and MDD in East Asians populations is crucial to evaluate its robustness and biological validity. Such research will be critical for developing tailored, ancestry-informed prevention strategies that bridge mental and cardiovascular health.”
17	**Generalizability**	Discuss the generalizability of the study results (a) to other populations, (b) across other exposure periods/timings, and (c) across other levels of exposure	23	Conclusion section<br><br>*Lines 465-472*<br><br>“This study provides evidence for a causal relationship between MDD and CAD, emphasizing the need to consider psychological factors in cardiovascular disease prevention and treatment. Although a preliminary inverse association between CAD and MDD was observed in East Asian populations, this finding did not meet corrected significance thresholds and was not supported by sensitivity analyses, suggesting that residual confounding or methodological biases may have influenced the result and that replication is required. Collectively, these results highlight the need for greater ancestral diversity in genetic research to ensure that causal inferences are robust and globally relevant, ultimately supporting more equitable public health strategies.”

	**OTHER INFORMATION**			
18	**Funding**	Describe sources of funding and the role of funders in the present study and, if applicable, sources of funding for the databases and original study or studies on which the present study is based	3	Funding section<br>*Lines 66-67*<br>“This research was entirely self-funded.”
19	**Data and data sharing**	Provide the data used to perform all analyses or report where and how the data can be accessed, and reference these sources in the article. Provide the statistical code needed to reproduce the results in the article, or report whether the code is publicly accessible and if so, where	23-24	Data Sharing Statement section<br>*Lines 476-480*<br>“The genome-wide association summary statistics data used in this study are publicly available at https://www.ebi.ac.uk/gwas/downloads/summary-statistics and https://pgc.unc.edu/for-researchers/download-results/. All software programs used in this paper are listed and referenced in the Methods. The full coding script used to perform the analysis is available from the corresponding authors upon request.”
20	**Conflicts of Interest**	All authors should declare all potential conflicts of interest	3	Declaration of Interests section<br>*Lines 62-63*<br>“Authors declare no competing interests.”

This checklist is copyrighted by the Equator Network under the Creative Commons Attribution 3.0 Unported (CC BY 3.0) license.

1. Skrivankova VW, Richmond RC, Woolf BAR, Yarmolinsky J, Davies NM, Swanson SA, et al. Strengthening the Reporting of Observational Studies in Epidemiology using Mendelian Randomization (STROBE-MR) Statement. JAMA. 2021;under review.

2. Skrivankova VW, Richmond RC, Woolf BAR, Davies NM, Swanson SA, VanderWeele TJ, et al. Strengthening the Reporting of Observational Studies in Epidemiology using Mendelian Randomisation (STROBE-MR): Explanation and Elaboration. BMJ. 2021;375:n2233.
